# A lactylation-ferroptosis cross-talk gene signature predicts hepatocellular carcinoma prognosis and reveals STMN1/PRDX1 as therapeutic targets

**DOI:** 10.3389/fimmu.2025.1677089

**Published:** 2025-12-01

**Authors:** Jiali Meng, Chunyan Liang, Ling Li, Qiqi Huang, Weimei Huang, Rensheng Wang, Xiaolong Li

**Affiliations:** 1 Department of Radiation Oncology, First Affiliated Hospital of Guangxi Medical University, Nanning, Guangxi, China; 2 Guangxi Key Laboratory of Immunology and Metabolism for Liver Diseases, Guangxi Medical University, Nanning, Guangxi, China; 3 Department of Pathology, the Sixth Affiliated Hospital, School of Medicine, South China University of Technology, Foshan, China; 4 Department of Cell Biology and Genetics, School of Pre-Clinical Medicine, Key Laboratory of Longevity and Aging Related Diseases of Chinese Ministry of Education, Guangxi Medical University, Nanning, China

**Keywords:** hepatocellular carcinoma (HCC), lactylation, ferroptosis, prognostic analysis, early-stage biomarkers

## Abstract

**Introduction:**

Hepatocellular carcinoma (HCC) presents great difficulties for diagnosis and prognosis. Metabolic reprogramming and ferroptosis resistance play critical roles in HCC development and progression.

**Methods:**

We combined transcriptome data from TCGA-LIHC to screen out lactylation-ferroptosis-related genes (LFRGs). Based on LASSO-Cox regression and multivariate analysis, a 4-gene-prognostic signature (STMN1, PRDX1, TP53, G6PD) was constructed.

**Results:**

The signature stratified HCC patients into two risk subgroups with obvious survival differences (P < 0.001). Functional validation demonstrated that STMN1/PRDX1 knockdown in MHCC-97H/SNU-449 cells could decrease the expression of LDHA (lactylation enzyme) and GPX4 (ferroptosis inhibitor), and further inhibited cell proliferation and migration (P < 0.01). The risk model was positively correlated with tumor stage and TMB, TP53 mutation, and immunosuppressive microenvironment. Drug sensitivity profile analysis suggested that high-risk patients may be sensitive to Dactolisib/Trametinib, while low-risk patients showed resistance to Axitinib/Ibrutinib. Mechanistically, STMN1/PRDX1 act as dual hubs in lactate metabolism (stabilizing LDHA) and ferroptosis resistance (modulating GPX4).

**Discussion:**

We identified the first lactylation-ferroptosis cross-talk signature to predict HCC prognosis and identified STMN1/PRDX1 as potential targets for treating HCC by stratifying therapies.

## Introduction

Hepatocellular carcinoma (HCC) exhibits considerable heterogeneity in clinical presentation and is associated with considerable economic burden. Being the sixth most common cancer and the third cause of cancer death worldwide (1), it accounts for almost all primary liver cancers and represents the most common histological type of liver cancer (2). The poor 5-year survival (less than 20%) in HCC patients is mainly due to its highly invasive property and acquired resistance to treatment in advanced stage. Early detection leads to improved survival in patients (3); however, there is a lack of sensitive biomarkers in clinical setting.

In the past few years, the cross-talk between metabolic reprogramming in the tumor microenvironment (TME) and novel cell death have been established as a crucial factor affecting hepatocellular carcinoma (HCC) progression, treatment resistance and immune escape (4). Its prognosis is poor, while the exact mechanistic cross-talks and pathological meanings between two potentially connected biological processes-lactylation and ferroptosis-in HCC remains systematically unexplored. Lactylation, also known as lysine lactylation (Kla), is a lactate-mediated post-translational modification. Its pathological significance in TME originates from the Warburg effect, in which neoplastic cells rely on glycolysis for energy production even in normoxic conditions (5, 6). Prominent glycolysis leads to high lactate production and further induces acidification of TME and high lactate contents. Acting as a signaling molecule, lactate regulates widespread lactylation modifications and participates in tumor progression in many ways. Research have revealed that lactate directly modifies lysine on histones and then influences the transcription of targeted genes. Notably, lactylation of histone H3K9 and H3K18 leads to the expression of M2-polarized macrophage genes and suppresses anti-tumor immunity (7). In HCC, lactylation has been documented to facilitate tumor proliferation, invasion/metastasis, angiogenesis, and immunosuppression, establishing itself as a critical epigenetic nexus bridging metabolic reprogramming with malignant phenotypic manifestations (8). Notably, lactate, the major metabolic product derived from glycolytic metabolism, regulates cancer cell death and modulates the TME by acting as an energy substrate and modulator; meanwhile, it engages in profound and poorly characterized functional cross-talks with another novel type of cell death. Ferroptosis is a novel type of regulated cell death, which is characterized by iron-dependent lipid peroxidation (9). Its role in cancer pathogenesis is dualistic. On one hand, cancer cells can exploit ferroptosis to eliminate certain tumor-suppressive components in the tumor-suppressive milieu. On the other hand, excessive ferroptosis has been demonstrated to serve as a natural tumor-suppression process that can inhibit tumor growth effectively (10, 11). This suppressive process is achieved by interacting with various tumor suppressor genes, such as p53 and BAP1, while many oncogenic mutations promote tumor development by conferring resistance to ferroptosis. Therefore, inducing ferroptosis in cancer cells provides a potential strategy for anticancer therapy (12, 13). Susceptibility to ferroptosis is intrinsically associated with cellular metabolic status. Perhaps surprisingly, the metabolic basis of ferroptosis is glycolysis, which is also the case with lactylation. Aberrant glycolytic activity and lactate overproduction may profoundly perturb ferroptosis sensitivity through: (i) Altering NADPH/GSH availability (critical cofactors for glutathione redox cycling); (ii) Disrupting iron homeostasis; or (iii) Directly modulating lipid synthase activity (14). Furthermore, lactylation functions as a dynamic epigenetic and signaling regulator that may directly modulate ferroptosis-associated genes such as GPX4 (15). Critically, recent studies greatly facilitate such integration; for example, lactylation of histone H3 at lysine 18 (H3K18la) enhances the expression of iron-sulfur cluster assembly protein NFS1 and reduces the susceptibility of HCC cells to ferroptosis and thus directly connects lactylation with ferroptosis resistance (16). Furthermore, this interplay exhibits striking spatial heterogeneity among distinct TME cellular compartments: Tumor cell-intrinsic lactylation dictates ferroptosis susceptibility (17); Tumor-derived lactate induces lactylation on immune cells (such as macrophages, T cells) and may switch their functional states (such as pro-/anti-inflammatory polarization) or induce their ferroptosis to directly modulate anti-tumor immune state and response to ferroptosis-inducing therapy (18). These findings collectively support our working hypothesis that this interplay constitutes a well-regulated lactylation-ferroptosis synergistic regulatory network whose central role in HCC progression, immune escape and therapy resistance is gradually emerging and thus we focused on this critical regulatory nexus in this study. By integrating multi-omics data from TCGA-LIHC, we cross-analyzed ferroptosis- and lactylation-related genes, identifying a core prognostic signature (STMN1, PRDX1, TP53, G6PD) through LASSO-Cox regression. Subsequent siRNA-mediated knock down of STMN1 and PRDX1 in MHCC-97h/SNU-449 cells (knockdown efficiency >70%, P<0.01) demonstrated that STMN1 suppression significantly downregulated LDHA (a key lactylation enzyme) and GPX4 (a ferroptosis inhibitor). Functional assays further confirmed that deficiency in either STMN1 or PRDX1 markedly impaired HCC cell migration and clonogenic capacity (all P<0.01). The established prognostic model provides a novel molecular tool for early-stage HCC stratification, offers a theoretical foundation for combinatorial targeting of the lactate metabolism-ferroptosis axis, and establishes a framework for developing innovative diagnostic biomarkers and therapeutic strategies.

## Materials and methods

### Information collection

Transcriptomic and clinical data from 377 HCC samples were obtained from The Cancer Genome Atlas (TCGA) database. A comprehensive list of 826 ferroptosis-related genes (FRGs) was curated from FerrDb V2 (http://www.zhounan.org/ferrdb/current/) (19). Based on prior established evidence, 308 lactylation-related genes (LRGs) were included (20). Intersecting LRGs and FRGs defined lactylation-ferroptosis-related genes (LFRGs). Functional enrichment of LFRGs was performed via Gene Ontology (GO) and KEGG pathway analyses using clusterProfiler (v3.14.3) (21), revealing their biological roles.

### Feature selection and model validation

Subsequently, LASSO-Cox regression analysis was performed on the intersection genes, from which multivariate Cox analysis identified four optimal prognostic markers (22) Their weighting coefficients were derived to construct the signature model. The risk score per patient was computed with the following formula: Risk score = ∑(i=1 to n)[Expr(gene_i)*Coef(gene_i)]. Here, Expr(gene_i) denotes the expression value of gene “i”, while Coef (gene_i) corresponds to its regression coefficient obtained from the multivariate Cox model; “n” = 4 indicates the number of genes in the signature. According to the training cohort median risk score, hepatocellular carcinoma (HCC) patients were further segmented into high- and low-risk subgroups. The Cox regression and KM survival curves were used to assess the independent prognostic value of the constructed risk signature. The nomogram was developed using the RMS package to predict overall survival (OS) probabilities. Model performance was evaluated by Harrell’s C-index, time-dependent ROC analysis and calibration curve. Finally, we analyzed the distribution of risk score in clinicopathological subgroups. To ensure rigorous validation, a two-stage approach was employed: 1) comprehensive assessment of the complete TCGA-LIHC cohort; 2) internal validation through equal partitioning (1:1) into training and test sets. Samples in all three cohorts were classified based on the median risk score, followed by generation of risk curves (comprising gene expression heatmaps, risk score distribution curves, and survival status dot plots) and Kaplan-Meier survival analysis.

### Correlation analysis of TME, TMB and immune checkpoints

Immune/stromal/ESTIMATE scores quantifying HCC TME components were computed via ESTIMATE algorithm (v1.0.13) (23). TMB was derived from TCGA-LIHC somatic mutations (mutations/Mb). Pearson correlation assessed risk score-TMB relationships using: 1) scatter plots (95% CI), 2) risk-stratified TMB boxplots (Wilcoxon test). Waterfall plots contrasting mutation profiles of high- versus low-risk cohorts were generated using maftools (R package). STMN1 and PRDX1 selection criteria derived from established literature evidence and our gene expression heatmaps of risk curves (24, 25). Furthermore, we conducted comprehensive analyses using R to investigate the specific associations between these two genes (STMN1 and PRDX1) and cancer pathogenesis.

### Immunotherapeutic drug sensitivity and efficacy profiling

Drug sensitivity prediction employed the “pRRophetic” R package, applying ridge regression to estimate half-maximal inhibitory concentrations (IC_50_) across sample cohorts for inferring chemotherapeutic response patterns (26). Therapeutic response disparities were assessed via Wilcoxon signed-rank testing of inter-group IC_50_ comparisons.

### Cell culture and transfection

MHCC-97H (ATCC CRL-8024) and SNU-449 (ATCC CRL-2234) cells were obtained from ATCC. MHCC-97H cells were cultured in DMEM (Gibco), SNU-449 in RPMI-1640 (Gibco), both supplemented with 10% FBS and 1% penicillin-streptomycin. All cells were maintained at 37°C, 5% CO2 and transfected at 80% confluency. For the knockdown experiments of STMN1 and PRDX1 genes, both cell lines were transfected using siRNA reagent kits from Shanghai GenePharma Co., Ltd. and Lipofectamine 3000 (Thermo Fisher Scientific, USA). The control group was transfected with siNC (negative control). RNA was extracted 24–48 hours post-transfection for qRT-PCR to assess transfection efficiency, while proteins were extracted approximately 72 hours after transfection for Western blot analysis.

### RNA and protein isolation coupled with qPCR and immunoblotting

Total RNA was isolated from cells using an extraction kit (NCM Biotech) per manufacturer’s protocol. cDNA synthesis employed a reverse transcription system (TaKaRa RR047A), followed by quantitative PCR (qPCR; TaKaRa RR420A) with 2^-ΔΔCt^ comparative threshold cycle analysis. Protein lysates from all experimental groups underwent immunoblotting with primary antibodies against: PRDX1, STMN1, LDH, GPX4 (HUABIO); GAPDH, α-Tubulin (Affinity).

### Cell counting kit-8 method

Following trypsinization, centrifugation, and counting, target cells were plated uniformly in 96-well plates at a density of 3×10³ cells per well with 100 μL medium. Wells with only culture medium and CCK-8 solution, devoid of cells, served as the blank control. The outer perimeter wells received 100 μL PBS to ensure humidity prior to 37°C incubation. After cell attachment, the plates were incubated for 0, 24, 48, and 72 hours. Subsequently, every well was supplemented with 10 μL CCK-8 solution and incubated for another 2 hours. Absorbance at 450 nm was measured on a microplate reader, followed by data processing. The experiment included three independent biological replicates, with each containing three technical repeats. Results are depicted in a line graph as the mean ± standard deviation from the biological replicates and were subjected to statistical evaluation using two-way ANOVA with multiple comparisons.

### Colony formation assay

Following a 14-day culture period in complete DMEM/RPMI-1640 media, the resulting cell colonies were subjected to processing. This involved fixation in 4% PFA for 15 minutes, subsequent staining with 0.5% crystal violet for 30 minutes, and final air-drying. Viable colonies, defined as those containing more than 50 cells, were manually counted. Data are presented as the mean ± standard deviation from three independent biological replicates.

### Wound healing assay and transwell migration assay

The MHCC-97H and SNU-449 cells were trypsinized, centrifuged, resuspended and counted separately. For the wound healing assay, both transfected cell types were seeded in 6-well plates at a density of 6×10^5^ cells per well. The cells were then incubated until attachment, after which wound scratches were created using a 200μL sterile pipette tip. After PBS rinses, serum-free medium was introduced before transferring plates to a 37°C incubator. Wound closure was periodically monitored (0/24/48h) with microscopic imaging, followed by ImageJ-based quantification. For the Transwell assay, Transwell chambers (8μm, 24-well inserts, Costar, USA) were used. Transfected cells (24h post-transfection) were plated in Transwell inserts, with the lower compartment containing 10% FBS medium as chemoattractant. Following migration, cells underwent methanol fixation and 0.1% crystal violet staining. Data from three independent biological replicates are presented as mean ± SD.

### Statistical analysis

All analyses were conducted in GraphPad Prism 10.1.2. Intergroup comparisons of continuous variables utilized Wilcoxon rank-sum and Kruskal-Wallis testing. Spearman’s ρ correlation coefficients were used to measure the association between variables. Multivariate Cox regression was used to evaluate the independent prognostic efficiency of LFRG-derived signature. Survival probabilities were calculated based on KM methodology. Statistical significance was set at two-sided P<0.05. ANOVA analysis and its *post hoc* test were used to analyze the transfection efficiency, proliferation level (CCK-8) and Transwell migration capacity. Two-way ANOVA with multiple comparisons was employed to assess the wound healing progression over time.

## Result

### Identifying LFRGs and conducting functional enrichment analysis

**Figure 1:**
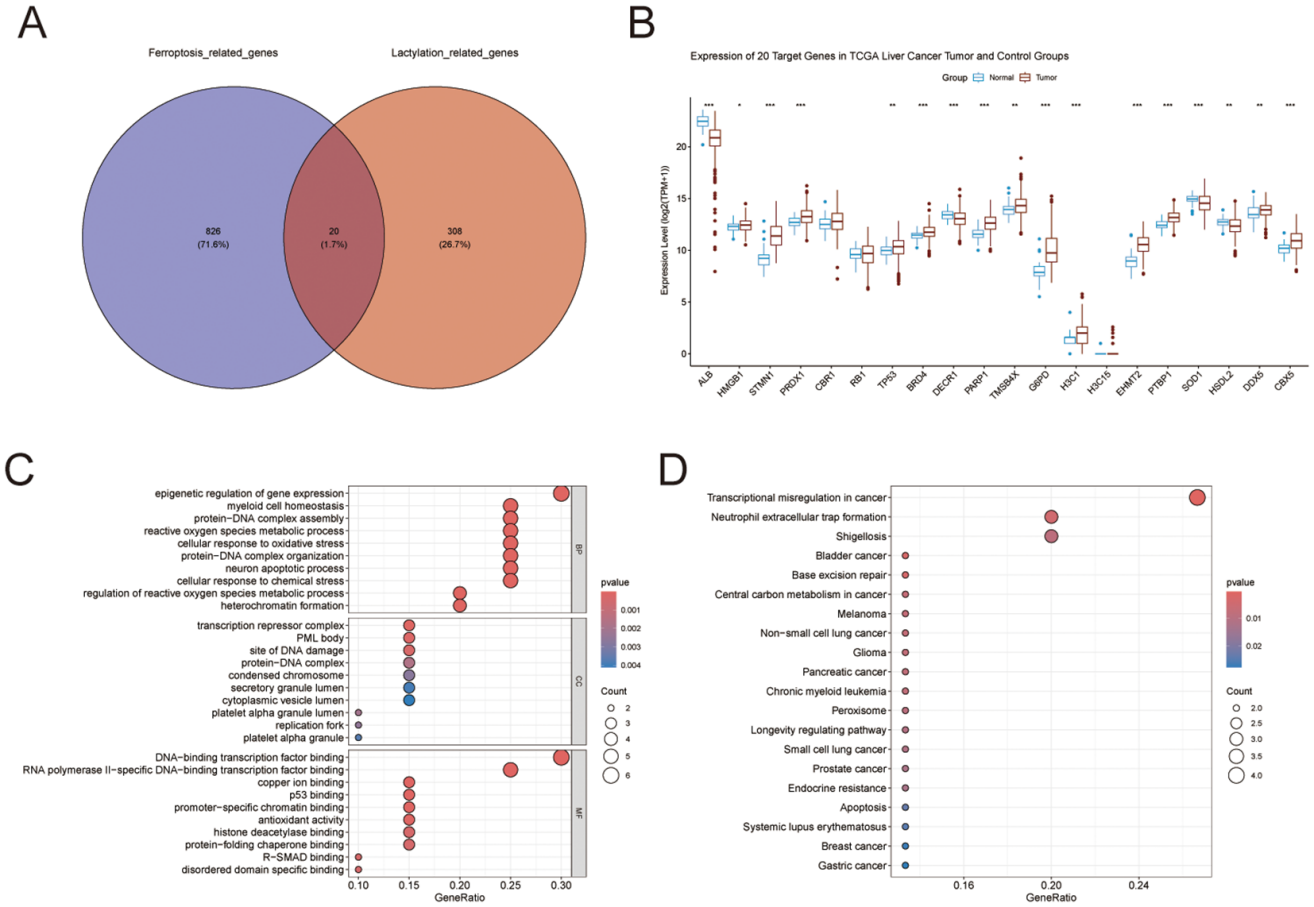
IIIdentification and functional annotation of LFRGs. **(A)** Identification of LFRGs. **(B)** Expression of LFRGs in HCC and normal tissues. **(C)** Gene Ontology (GO) functional enrichment. **(D)** Pathway enrichment mapping via KEGG. *p < 0.05, **p < 0.01, ***p < 0.001.

Intersecting 308 LRGs with 826 FRGs revealed 20 LFRGs (**Figure 1A**). **Figure 1B** displays the expression profiles of these 20 genes in HCC versus normal tissues; notably, STMN1, PARP1, G6PD, EHMT2 and several others exhibited significantly elevated expression in HCC. GO enrichment results are presented in **Figure 1C**. Key biological processes involved: epigenetic gene expression regulation, myeloid cell homeostasis, protein-DNA complex assembly, reactive oxygen species metabolism, cellular response to oxidative stress, protein-DNA complex organization, neuronal apoptosis, and cellular response to chemical stress. The cellular component categories included transcription repressor complex, PML body, site of DNA damage, protein−DNA complex, condensed chromosome and secretory granule lumen. The molecular function categories included DNA−binding transcription factor binding, RNA polymerase II−specific DNA−binding transcription factor binding, copper ion binding, p53 binding, promoter−specific chromatin binding, antioxidant activity, histone deacetylase binding and protein−folding chaperone binding (**Figure 1C**). KEGG analysis showed Transcriptional misregulation in cancer, Neutrophil extracellular trap formation, Shigellosis, Bladder cancer, Base excision repair and Central carbon metabolism in cancer (**Figure 1D**).

### Identification of LFRGs and their prognostic correlation

**Figure 2:**
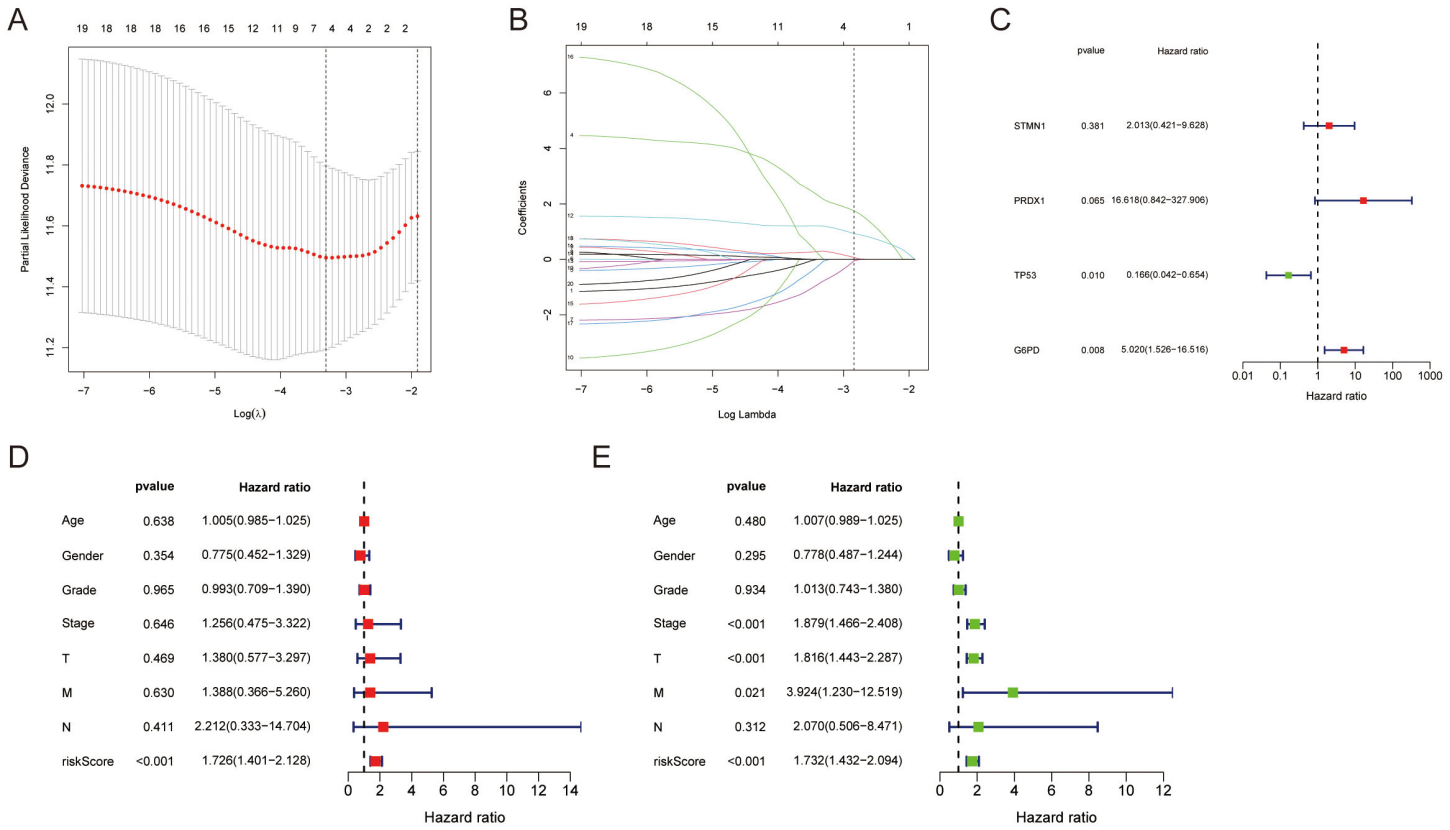
Prognostic biomarker evaluation of LFRGs in hepatocellular carcinoma **(A)** LASSO coefficient trajectories showing optimal λ selection via cross-validation. **(B)** Regularization paths depicting feature stability across λ values. **(C)** Multivariable Cox regression confirming independent prognostic impact of core genes (STMN1/PRDX1/TP53/G6PD). **(D, E)** Integrated clinical-genomic model: Identified stage and risk score as survival determinants; Dichotomized patients by median risk score.

In the TCGA-LIHC cohort of 424 HCC patients, 20 candidate genes related to LFRGs were identified through intersectional analysis. LASSO regression narrowed these down to 19 potential prognostic genes (**Figures 2A, B**). Ultimately, multivariable Cox modeling confirmed four key biomarkers: STMN1, PRDX1, TP53, and G6PD (**Figure 2C**). After adjustment for clinical variables including age, sex, and tumor stage, these genes constituted principal independent predictors of overall survival (OS) in HCC patients. A prognostic model incorporating STMN1, PRDX1, TP53, and G6PD expression levels was developed, with risk scores calculated from these values. Stratification into high- and low-risk groups was performed using the median risk score as cutoff (**Figure 2D**). Significantly worse OS rates were observed in the high-risk versus low-risk cohort (p<0.001), confirming the LFRGs signature’s prognostic value. Clinical variables, such as this gene signature and tumor stage, were examined through univariate and multivariate Cox regression analyses. Both analyses consistently identified the gene signature (HR = 2.41, 95% CI 1.87-3.11) and tumor stage (HR = 1.89, 95% CI 1.42-2.52) as independent predictors of survival outcomes in HCC patients (**Figure 2E**). Based on the relationships of STMN1, PRDX1, TP53 and G6PD with lactate metabolism, ferroptosis and survival, our analysis confirmed these four genes as key prognostic biomarkers for hepatocellular carcinoma. This four-gene prognostic model reliably distinguished high- and low-risk HCC patients, demonstrating clinical utility for precision medicine approaches.

### Establishment and verification of risk score distribution and prediction nomogram

**Figure 3:**
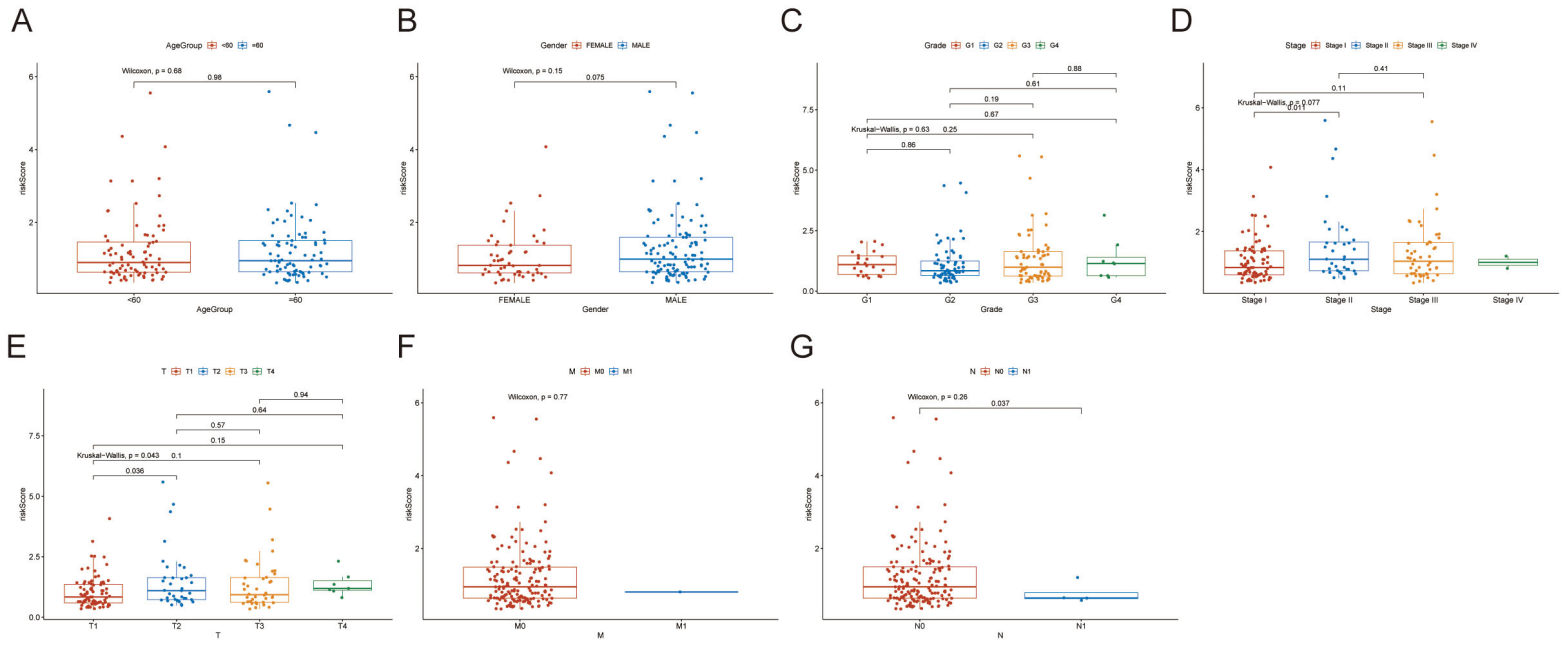
Risk score distribution across HCC subgroups by age, gender, WHO grade, stage, and TNM stage. **(A)** Age Group. **(B)** Gender. **(C)** WHO Grade. **(D)** Stage. **(E)** T Stage. **(F)** M Stage. **(G)** N Stage.

**Figure 4:**
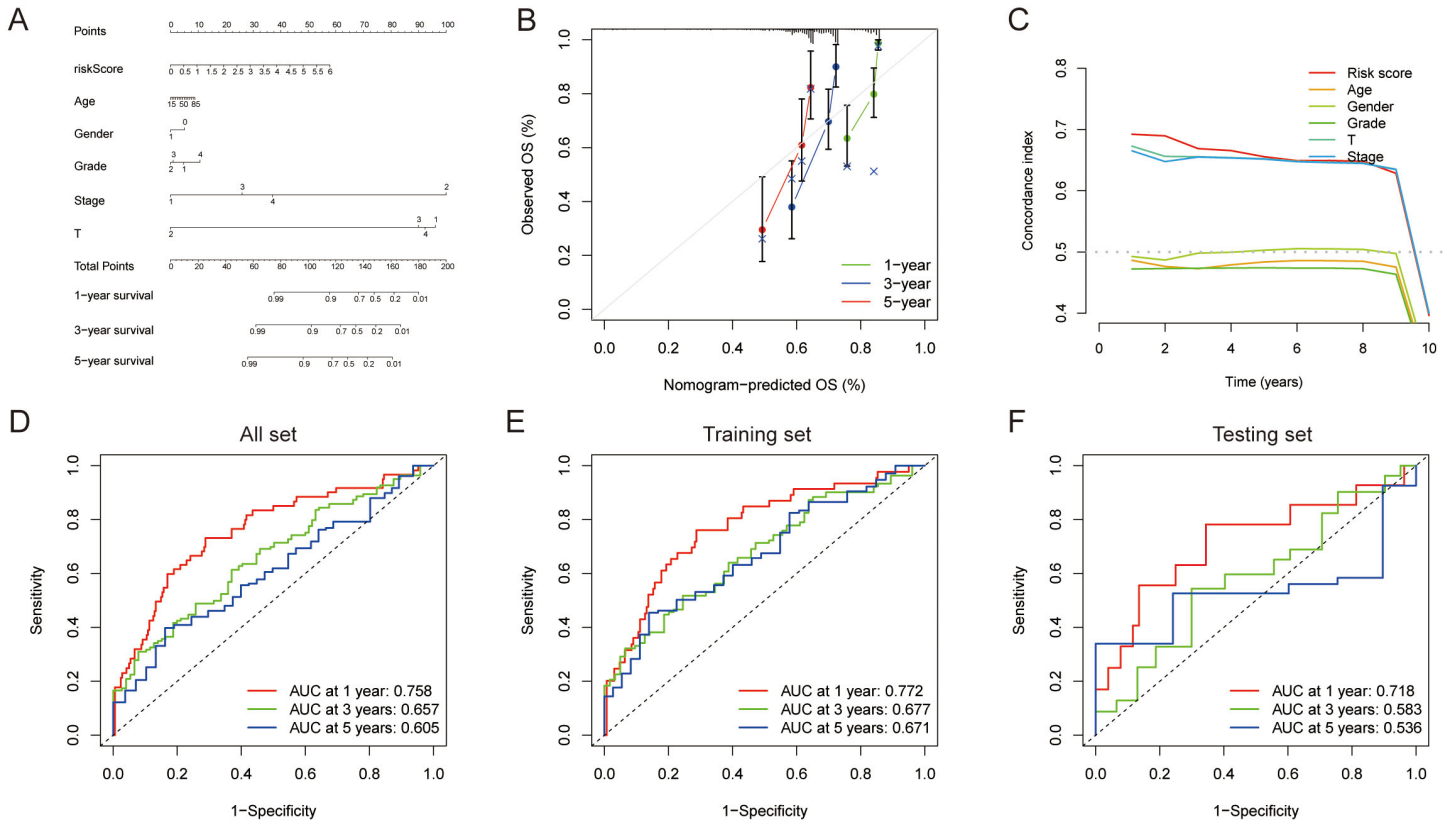
Nomogram model combining risk score and clinical parameters. **(A)** Nomogram for predicting 1-year, 3-year, and 5-year OS rates in HCC patients. **(B)** Calibration curve of the nomogram model. **(C)** The concordance index plot illustrates the independent prognostic value of risk score, age, gender, grade, T stage, and overall stage over 1–10 years. **(D-F)** Time-dependent ROC curves of three sets evaluating risk score accuracy in predicting survival at 1-, 3-, and 5-year intervals.

Risk score distributions demonstrated differential patterns across clinical subgroups (**Figure 3**), with statistically significant disparities observed between T1 and T3 stages, between Stage I and Stage II and between N0 and N1 (risk scores exhibited stepwise elevation with advancing stages). We developed a prognostic nomogram using the TCGA cohort to predict survival probabilities in HCC patients by incorporating six independent prognostic factors: riskScore, Age, Gender, Grade, Stage, and T stage, to estimate overall survival (OS) probabilities at specified timepoints (**Figure 4A**). The 45-degree diagonal represented the ideal reference line, while calibration curves confirmed the nomogram’s precision for 1-, 3-, and 5-year survival outcomes (**Figure 4B**). Concordance index analysis revealed risk score, T-stage, and overall stage as robust survival predictors, maintaining sustained high C-index values. Receiver operating characteristic (ROC) curves of three sets further validated the model’s predictive accuracy for 1-, 2-, and 3-year OS: AUC values consistently surpassed 0.65 across all cohorts, with several exceeding 0.7 (**Figures 4D-F**). These findings underscore our model’s clinical utility for precise OS prediction and its translational promise in long-term HCC survival prognostication.

### Construction and validation of prognostic models for genes related to lactic acid and ferroptosis

**Figure 5:**
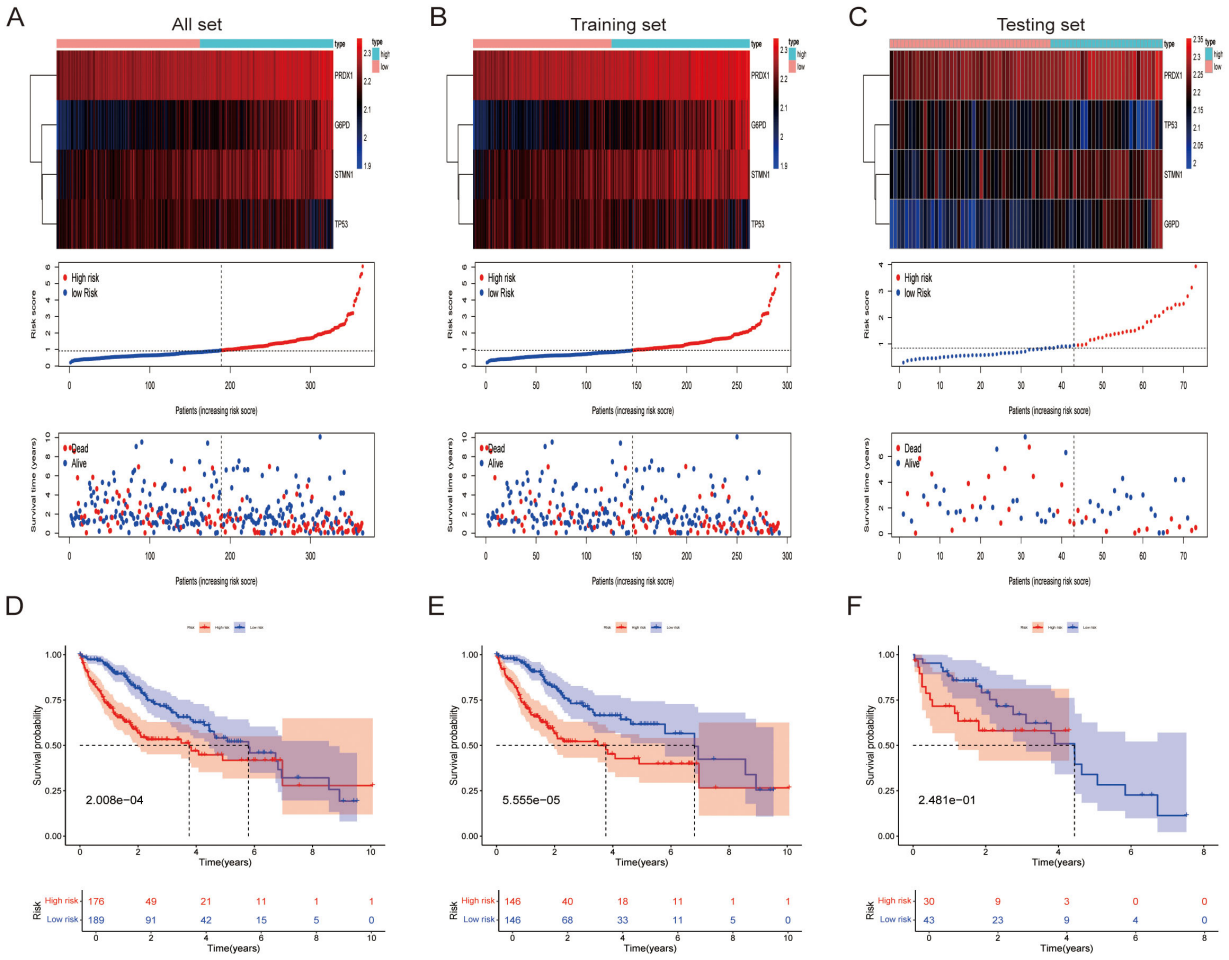
Validation of the risk score. **(A-C)** The risk curves consist of gene expression heatmaps, risk score distribution curves, and survival status dot plots. **(D)** For the entire cohort, OS diverged markedly between risk strata (Kaplan–Meier, χ² test, p = 2.008e-04). **(E)** Similar separation emerged within the training subset (χ², p = 5.555e-05). **(F)** The testing subset likewise exhibited pronounced risk-dependent OS differences (χ², p = 2.481e-1).

We developed and validated a lactate-ferroptosis-related gene (LFRG) prognostic signature (STMN1, PRDX1, TP53, G6PD) using the TCGA-LIHC cohort, evaluated across the full dataset (**Figures 5A, D**), internal training subset (**Figures 5B, E**), and testing subset (**Figures 5C, F**). Risk categorization revealed significantly diverging survival (P<0.001) between high- and low-risk groups in the full cohort, with elevated mortality and reduced overall survival (OS) corresponding to increasing risk scores (**Figure 5D**). Integrated visualization of risk distributions and expression heatmaps (**Figures 5A-C**) demonstrated differential upregulation of PRDX1 and STMN1 in high-risk patients, while G6PD/TP53 showed comparable expression, confirming adverse outcome associations. Both training and testing subsets consistently replicated prognostic stratification (**Figures 5E, F**) with conserved expression patterns (**Figures 5B, C**), establishing reproducible prognostic utility. These findings designate LFRGs as clinically relevant prognostic indicators for hepatocellular carcinoma.

### Associations between features and immune cell infiltration, mutation burden, immune cells, and immune function

**Figure 6:**
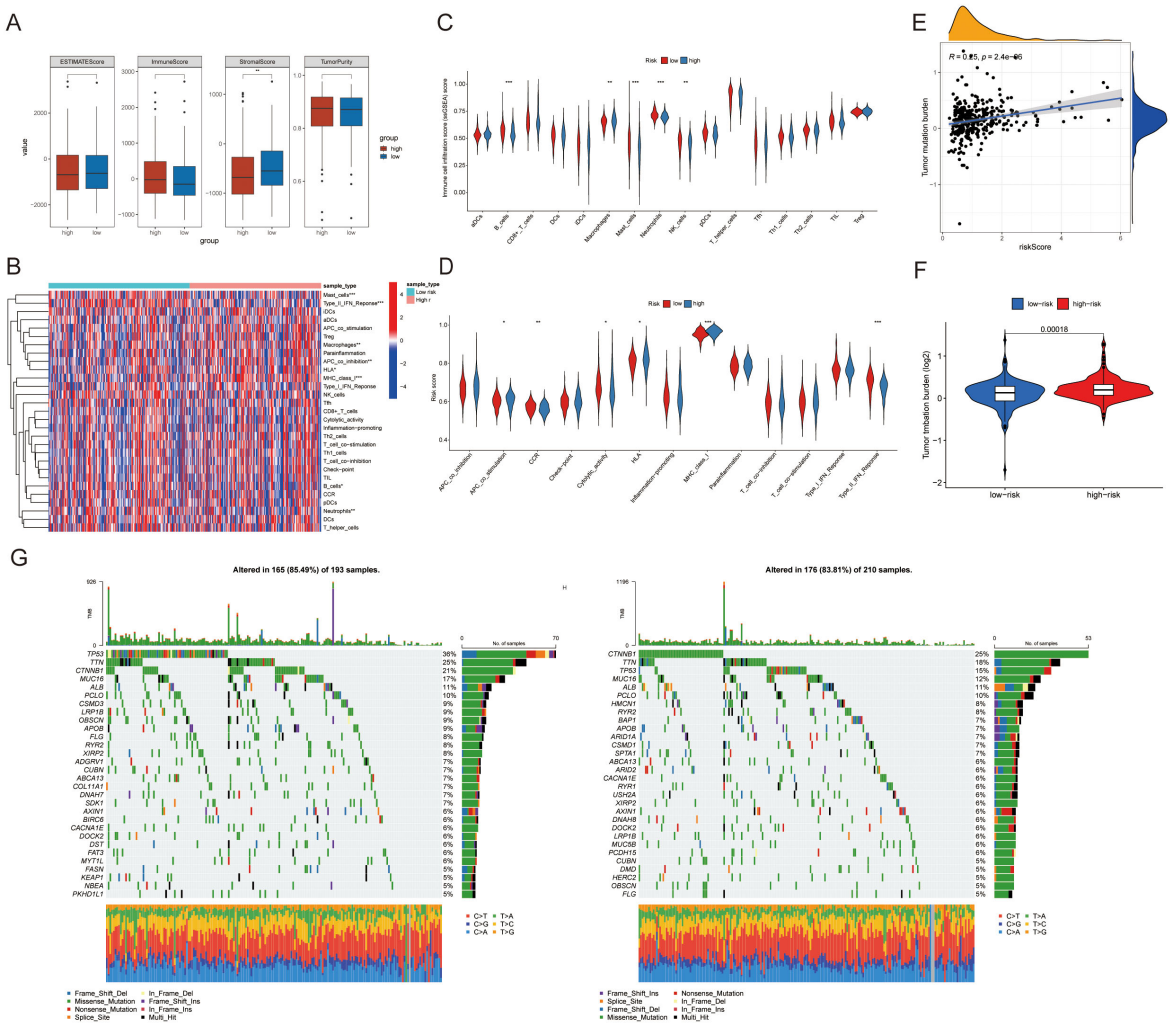
Evaluation of tumor microenvironment, immune cells/functions, and tumor mutation burden (TMB) across different groups. **(A)** Comparison of ESTIMATE scores, stromal scores, immune scores, and tumor purity between the two groups. **(B)** Comparison of immune cell infiltration between the two risk groups. **(C, D)** Differential expression of immune cell infiltration and immune functions between high-risk and low-risk groups. **(E)** Tumor mutation burden between high-risk and low-risk groups. **(F)** Differential analysis of TMB between high-risk and low-risk groups. **(G, H)** Somatic mutation waterfalls stratify patients by risk: every vertical track denotes one case. TMB appears in the overhead bar chart, right-side numerals quantify per-gene alteration rates, and the lateral bar graph depicts variant-class fractions. *p <0.05; **p<0.01; ***p<0.001; ****p<0.0001.

As a landscape-altering modality, cancer immunotherapy has substantially extended overall survival (OS) in patients with malignancies (27). Nevertheless, the density of tumor-infiltrating lymphocytes (TILs) and expression profiles of immune checkpoint molecules critically govern therapeutic responses to cancer immunotherapy (28, 29). Consequently, we systematically evaluated immune correlates of the prognostic signature. ESTIMATE scores, immune scores, and tumor purity were comparable across risk groups, whereas stromal scores demonstrated significantly elevation in low-risk cohorts relative to high-risk counterparts (**Figure 6A**). Subsequently, we examined associations among these four key genes and immune cells/functions. The immune-related correlation heatmap (**Figure 6B**) summarizes these findings. Significant disparities in immune cell composition and functional activity emerged between high- versus low-risk samples. Notably, high-risk samples displayed heightened immune activation along with cytotoxic activity, while low-risk counterparts manifested stronger regulatory and suppressive immune functions. Further comparative analysis of immune cell and functional expression patterns across risk groups was conducted. Violin plots confirmed statistically marked variations in immune cell infiltration, including B cells, macrophages, mast cells, neutrophils, and NK cells (**Figure 6C**), as well as immune functions (namely APC co-stimulation, CCR, cytolytic activity, HLA, and MHC class) (**Figure 6D**). Accumulating evidence implicates cancer stem cells (CSCs) as fundamental drivers of tumor relapse, metastatic dissemination, and chemoresistance development (30). Therefore, we performed stemness-associated analyses. The correlation scatter plot (**Figure 6E**) demonstrated that higher risk scores were associated with elevated stemness indices. Compared with the low-risk subset, the high-risk subset exhibited markedly elevated TMB (**Figure 6F**). We then contrasted mutational landscapes between these strata; waterfall plots (**Figures 6G, H**) showed TP53 alterations were strikingly enriched in the high-risk subset.

### The signature predicts efficacy of chemotherapy and immunotherapy

**Figure 7:**
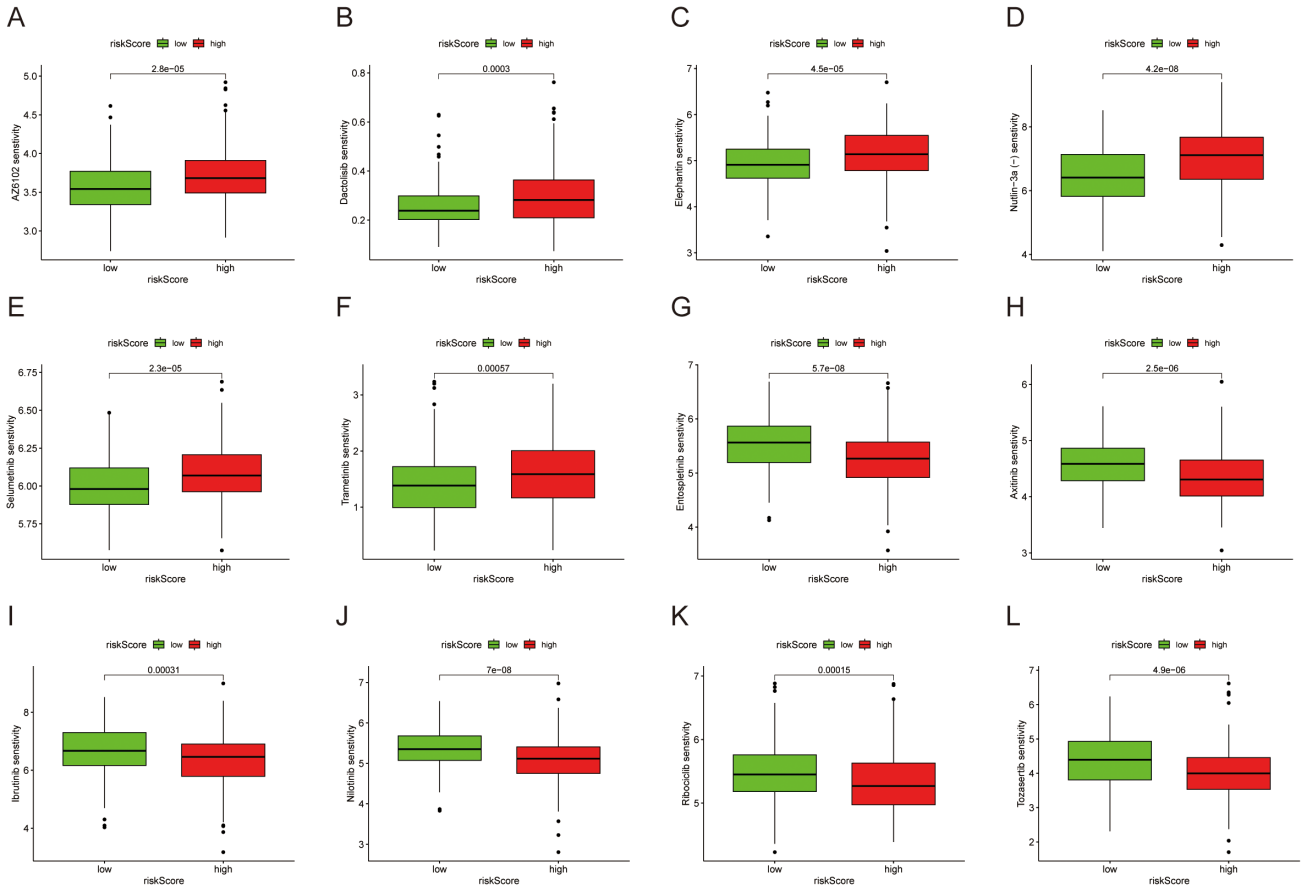
Analysis of differences in IC_50_ values of common drugs or compounds. **(A)** AZ6102. **(B)** Dactolisib. **(C)** Elephantin. **(D)** Nutlin-3a(-). **(E)** Selumetinib. **(F)** Trametinib. **(G)** Entospletinib. **(H)** Axitinib. **(I)** Ibrutinib. **(J)** Nilotinib. **(K)** Ribociclib. **(L)** Tozasertib.

To gauge whether the signature forecasts HCC drug response, we contrasted therapeutic outcomes between high- and low-risk subsets. Tumors in the low-risk subset exhibited elevated IC_50_ for Axitinib, Entospletinib, Ibrutinib, Nilotinib, Ribociclib and Tozasertib (**Figures 7G-L**), whereas the high-risk subset proved markedly more susceptible to AZ6102, Dactolisib, Elephantin, Nutlin-3a(-), Selumetinib and Trametinib (**Figures 7A-F**).

### Characteristic analysis of STMN1 and PRDX1 genes

**Figure 8:**
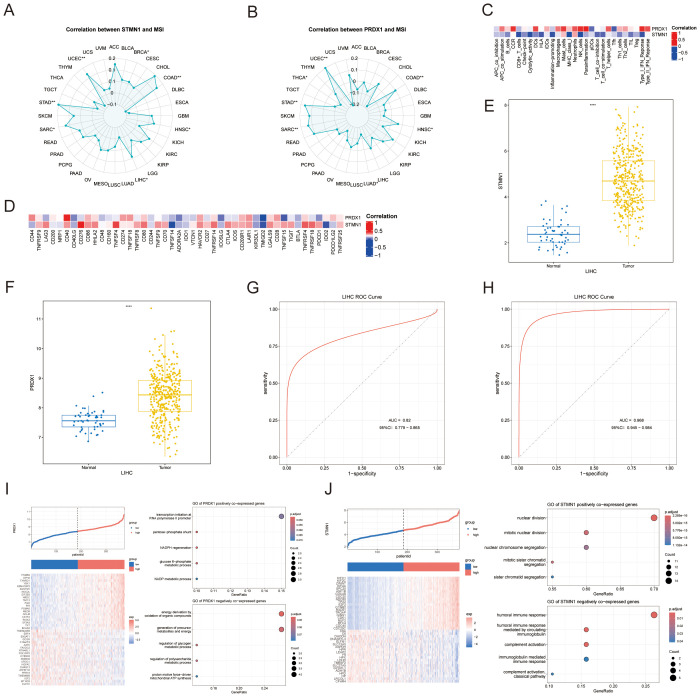
Assess the relationship between STMN1 and PRDX1 and cancer and the characteristics of single genes. **(A, B)** Correlation between STMN1/PRDX1 and MSI. **(C)**Heat map of the correlation between target genes and immune cells. **(D)**Heat map of correlation between target genes and immune checkpoints. **(E, F)** Target genes are expressed in normal tissues and liver cancer. **(G, H)** ROC curve of target gene for liver cancer prediction, and the area under the curve represents predictive power. **(I, J)** Single-gene characteristics of target genes in high-risk and low-risk groups include gene expression in high-risk groups, heat map of co-expressed genes, and GO terms in which positive and negative co-expressed genes mainly participate, *p <0.05; **p<0.01; ***p<0.001; ****p<0.0001.

Microsatellite instability (MSI) is a marker of genomic instability. The expression levels of STMN1 and PRDX1 genes are significantly correlated with MSI status in many cancers, and the strength of the correlation varies depending on cancer type. There is such a relationship between STMN1 gene and liver cancer (**Figure 8A**), while PRDX1 is most correlated with endometrial cancer (**Figure 8B**). Spearman correlation analysis revealed distinct associations of STMN1 and PRDX1 with immune features. PRDX1 demonstrated significant positive correlations with immune cells such as NK cells, para-inflammation, and mast cells, whereas STMN1 was significantly negatively correlated with CD8+ T cells, MHC class I, and NK cells (**Figure 8C**). At the level of immune checkpoints, PRDX1 correlated positively with CD40 but negatively with TMIGD2. In contrast, STMN1 expression was positively associated with CD276 and negatively associated with both TNFSF14 and TMIGD2 (**Figure 8D**). Collectively, these correlation analyses suggest that PRDX1 and STMN1 are distinctly associated with different components of the immune microenvironment in HCC. Boxplot reveals marked up-regulation of both genes in hepatocellular carcinoma (**Figures 8E, F**); ROC analysis further confirms their outstanding predictive value for HCC patients (**Figures 8G, H**), reinforcing our prognostic model’s reliability. Next, we will further explore the value of PRDX1 and STMN1 in the high and low risk groups we identified in the previous period (**Figures 8I, J**). Functional annotation revealed distinct roles for STMN1 and PRDX1 co-expressed genes: STMN1-associated genes primarily engaged in chromosomal segregation processes, while PRDX1 partners were enriched in transcriptional activation events mediated by RNA polymerase II. The GO terms in which the negative co-expressed genes mainly participate are humoral immune response and energy derivation by oxidation of organic compounds respectively.

### Suppression of malignant phenotypes in hepatocellular carcinoma cells following target gene silencing

**Figure 9:**
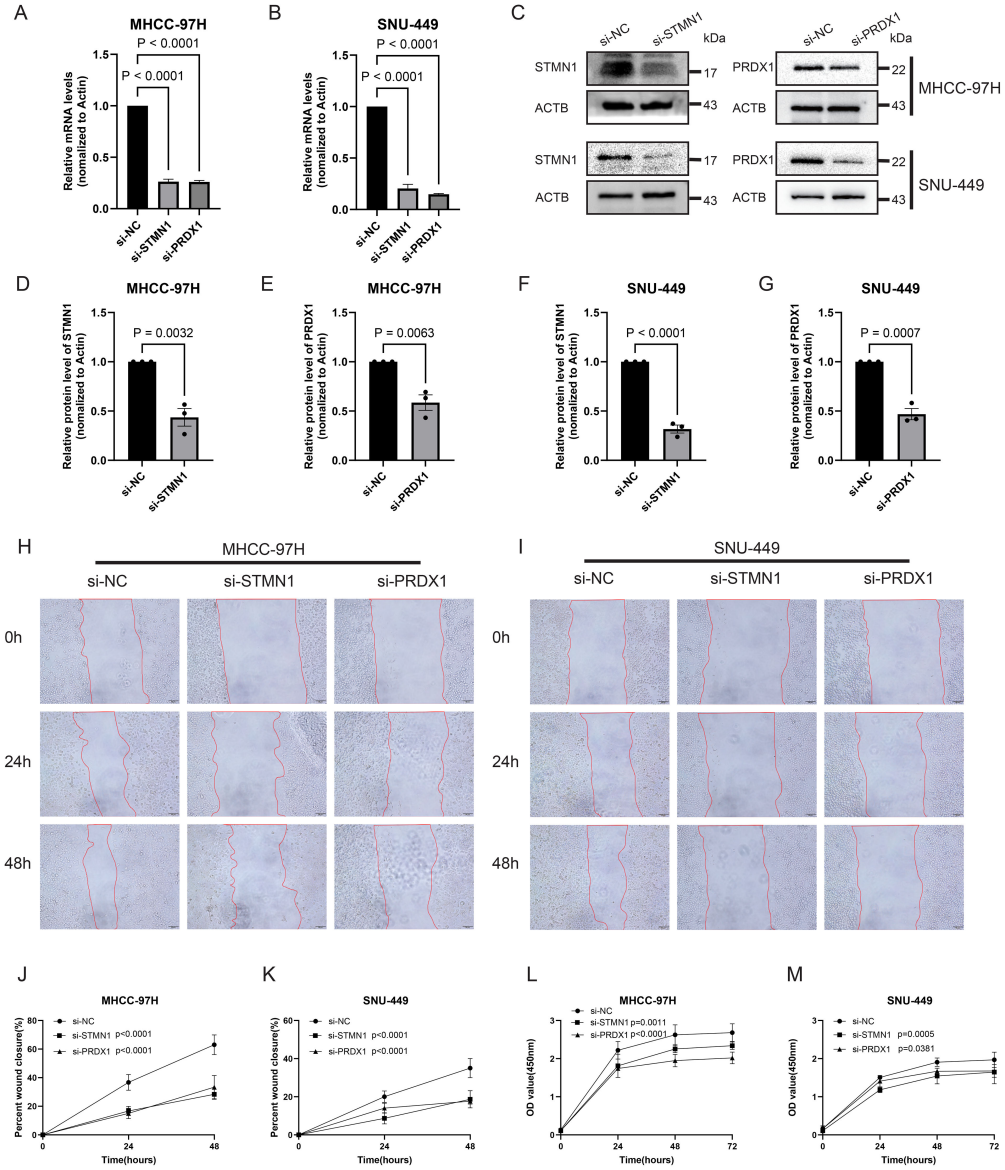
Cell experimental results of gene silencing. **(A-G)** Verification of knockdown efficiency of MHCC-97H and SNU-449 cells using qPCR and WB. **(H-K)** Wound healing experiments showed that MHCC-97H and SNU-449 cells knocked down by STMN1 and PRDX1 showed delayed wound healing compared to the si-NC group. **(L, M)** After knocking down STMN1 and PRDX1, the proliferation ability of MHCC-97H and SNU-449 was significantly inhibited in the cck8 assay. *p <0.05; **p<0.01; ***p<0.001; ****p<0.0001.

**Figure 10:**
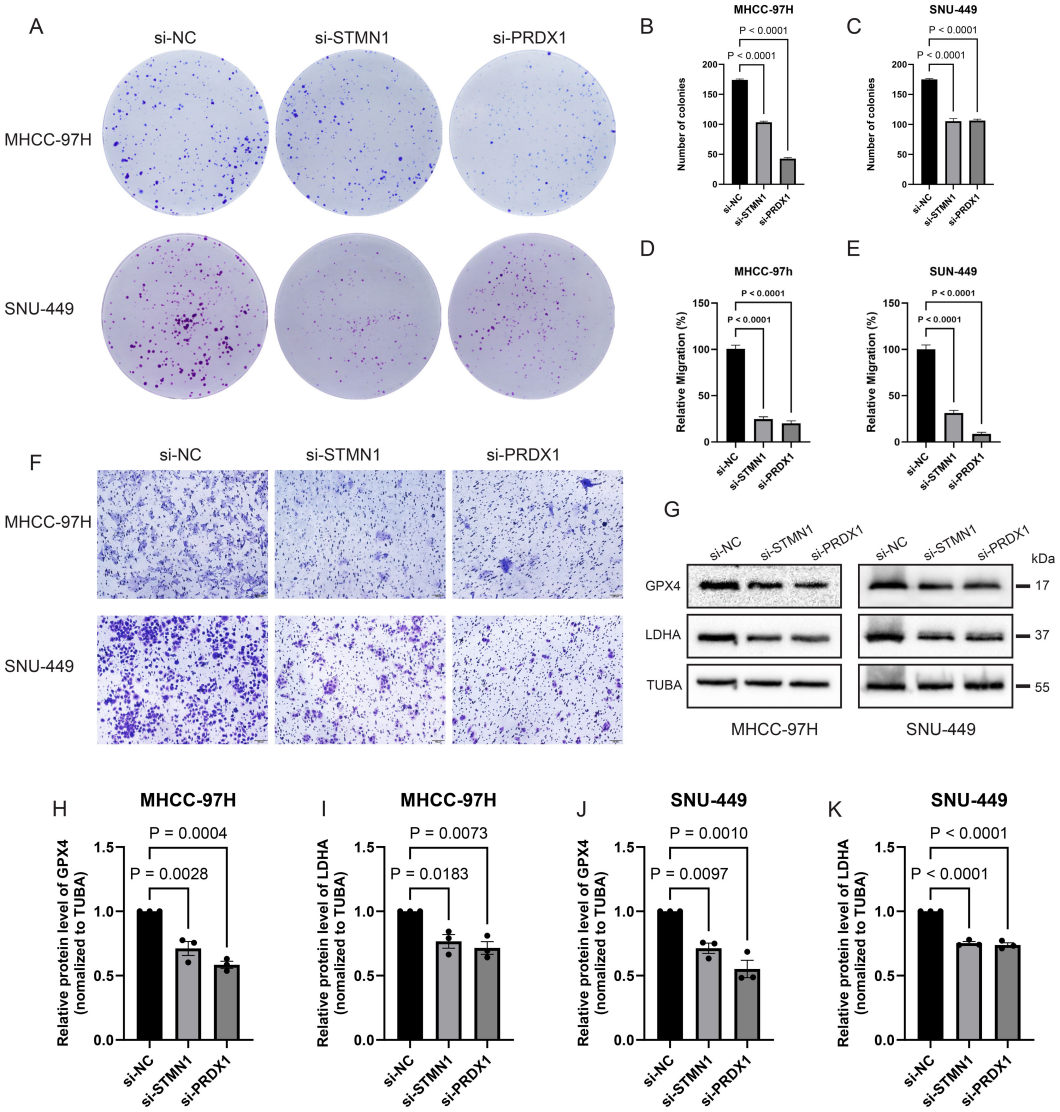
STMN1/PRDX1 knockdown inhibits HCC cell colony formation, migration, and reduces LDHA/GPX4 expression. **(A-C)** Colony formation of MHCC-97H and SNU-449 cells with or without STMN1 and PRDX1 knockdown. **(D-F)** Migration experiments showed that the migration ability of MHCC-97H and SNU-449 was inhibited after knocking down STMN1 and PRDX1. **(G-K)** WB Analysis showed that after STMN1 and PRDX1 knockdown, LDHA protein and GPX4 protein levels decreased in MHCC-97H and SNU-449 cells. *p <0.05; **p<0.01; ***p<0.001; ****p<0.0001.

Based on the results in **Figures 6**, **7** and relevant literature research (31–33), we selected two genes, PRDX1 and STMN1, as the main objects for our subsequent experimental verification. We ordered siRNA lentiviruses corresponding to two genes to knock down two key genes in liver cancer cells. The qPCR and WB results showed that the knockdown efficiency of the lentivirus was higher than 70%, proving that the lentivirus knockdown is effective (**Figures 9A-G**). CCK-8 viability assays and colony formation tests were employed to evaluate target gene effects on hepatocellular carcinoma (HCC) cell proliferation. To quantify the biological impact, we calculated the percentage reduction in cell viability. At the 72-hour time point, knockdown of STMN1 and PRDX1 resulted in an average decrease of 12.8% and 24.7%, respectively, in MHCC-97H cells compared to the si-NC control (**Figures 9L, M**). This anti-proliferative effect was consistently observed in colony formation assays, where the number and size of colonies were markedly reduced in the knockdown groups (**Figures 10A-C**).

To quantitatively assess the impact of STMN1 and PRDX1 on HCC cell motility, we first performed wound healing assays to evaluate collective cell migration. Genetic silencing of either gene significantly impeded wound closure in both MHCC-97H and SNU-449 cell lines. After 48 hours, the relative wound closure in MHCC-97H cells was markedly reduced to approximately 28% in the si-STMN1 group and 33% in the si-PRDX1 group, compared to the si-NC control (set as 100%). A consistent inhibitory effect was observed in SNU-449 cells, where the wound closure rates were only 18% (si-STMN1) and 17% (si-PRDX1) (**Figures 9J, K**). The impairment of migratory capacity was further confirmed and quantified using Transwell assays, which measure the potential for traversing an extracellular matrix-like barrier. The results were entirely concordant with the wound healing data. In MHCC-97H cells, the relative migration rates plummeted to approximately 25% upon STMN1 knockdown and to 20% upon PRDX1 knockdown. Similarly, in SNU-449 cells, migration was suppressed to 31% (si-STMN1) and 9% (si-PRDX1) of the control level (p < 0.0001 for all; **Figures 10D–F**).

Collectively, the quantitative data from these two independent functional assays—demonstrating consistent and severe defects in both collective sheet movement and individual cell invasion—provide compelling evidence that STMN1 and PRDX1 are critical drivers of HCC cell migration.

In order to further clarify the relationship between target genes and lactate metabolism and ferroptosis, we selected LDHA, a key enzyme in lactate metabolism, and GPX4, a classical pathway of ferroptosis, for WB verification. The results showed that when the expression of PRDX1 and STMN1 was knocked down, the expression of key genes in the two pathways also decreased (**Figures 10G-K**). Cell experimental results demonstrated that STMN1/PRDX1 knockdown inhibits LDHA (lactate metabolism pathway) and GPX4 (ferroptosis pathway), weakening cell proliferation, migration and other malignant phenotypes.

## Discussion

This study established for the first time a cross-regulatory network between lactic acid metabolism and ferroptosis pathways in hepatocellular carcinoma by integrating multiple genomic data from the TCGA database. Our study highlights the identification of genes associated with the intersection of several lactic acid metabolism and ferroptosis gene sets, particularly STMN1, PRDX1, TP53 and G6PD, forming a core prognostic signature. The constructed 4-gene risk model can prioritize stratification of patients. Our study particularly emphasizes the function of STMN1 and PRDX1 as key drivers in hepatocellular carcinoma progression.

The selection of STMN1 and PRDX1 for experimental validation was based on their well-documented roles in HCC and compelling links to the processes underpinning our study. STMN1 (Stathmin 1) is a cytosolic phosphoprotein that regulates microtubule dynamics, thereby controlling mitotic spindle formation and cell motility. Its overexpression is a hallmark of aggressive HCC, correlating with advanced tumor stage, microvascular invasion, and poor prognosis (31). PRDX1 (Peroxiredoxin 1) is a fundamental antioxidant enzyme that scavenges hydrogen peroxide and other peroxides to maintain cellular redox balance. Its upregulation in HCC promotes tumorigenesis by inhibiting apoptosis and facilitating pro-tumorigenic autophagy (24, 34). Critically, both genes have been independently implicated in ferroptosis: STMN1 via a specific transcriptional program discussed below, and PRDX1 by virtue of its central role in combating peroxidative stress, with its inhibition known to induce ferroptosis (33).

Our functional investigations reveal that these two genes are central to the lactylation-ferroptosis cross-talk. Experimental evidence demonstrates that knocking down either STMN1 or PRDX1 suppresses the expression of LDHA (a key enzyme for lactate metabolism) and GPX4 (a core regulator of ferroptosis), markedly reducing HCC cell malignant behaviors including proliferation, migratory capacity, and invasiveness. It was also found that high-risk tumors present a characteristic immune microenvironment: although there are cytotoxic phenotypes such as enhanced CD8 T cell infiltration, adaptive immune resistance driven by high-frequency mutations in TP53 (**Figures 6G, H**) and accompanying genomic instability (TMB↑) and enhanced tumor stemness (**Figure 6E**) lead to significant deterioration in survival outcomes.

Regarding the STMN1 gene, studies have shown that STMN1 is a biomarker for diagnosing microvascular infiltration, may be a potential therapeutic target for inhibiting HCC metastasis, and is also involved in the regulation of immune infiltration and M6A methylation in HCC (31). Furthermore, beyond its roles in diagnosis and metastasis, STMN1 expression is transcriptionally upregulated by the centromere protein CENPA, which binds to the STMN1 promoter to drive its expression (32). This CENPA-STMN1 axis constitutes a key regulatory network that inhibits ferroptosis, thereby promoting HCC growth (32). Our findings, which suggest the involvement of a CENPA-STMN1/GPX4 pathway, strongly support and extend this established mechanistic understanding. Not only that, STMN1 can also serve as a target for miR-101 to inhibit cisplatin-mediated autophagy in HCC cells (35). Collectively, numerous investigations indicate a strong association between the STMN1 gene and hepatocellular carcinoma development, positioning it as a potential diagnostic/prognostic biomarker. For another gene, PRDX1 is a gene encoding peroxide oxide protein with the same name. Evidence indicates that Celastrol, a bioactive triterpene derived from Tripterygium wilfordii, specifically targets the reactive cysteine residue of PRDX1, suppressing its antioxidant function. This suppression elevates intracellular ROS, subsequently triggering ferroptosis (33). Concurrently, research demonstrates that PRDX1 promotes hepatocellular carcinoma development through suppression of the intrinsic mitochondrial apoptosis pathway. Furthermore, PRDX1 facilitates hepatocyte autophagy, contributing to tumor advancement (34, 36). Collectively, mounting evidence identifies STMN1 and PRDX1 as potential biomarkers for hepatocellular carcinoma (HCC), further establishing their association with ferroptosis. Recent findings elucidate a direct link between histone lactylation and ferroptosis. Specifically, the lactate-induced histone mark H3K18la enhances the transcription of NFS1, a key enzyme in iron-sulfur cluster assembly. The subsequent upregulation of NFS1 confers resistance to ferroptosis in HCC cells (16). But the key hub connecting the two has not yet been reported.

Our study provides novel evidence that the two are co-regulated by lactic acid metabolism. Integrating findings from gene silencing experiments (**Figure 9**) and pathway enrichment analysis (**Figures 1C, D**), GO analysis revealed significant enrichment of FLRGs in the “promoter specific chromatin binding” term (**Figure 1C**), indicating that lactylation potentially directly activates STMN1/PRDX1 transcription. There may be a positive metabolic feedback cycle between the two, that is, STMN1 promotes LDHA mitochondrial translocation by stabilizing the microtubule structure; PRDX1 binds to LDHA and maintains its stability, accelerating lactate production; and finally forms a cross-link in the ferroptosis pathway, that is, PRDX1 protects GPX4 activity through antioxidant functions and forms a “LDHA-PRDX1-GPX4” complex, which together acts to resist ferroptosis. For clinical applications, a combination test of plasma lactic acid or hydroxythiolysine can be tried to improve early diagnosis rates. Advanced patients may benefit from using a combination regimen of LDHA inhibitors and ferroptosis inducers.

Utilizing lactic acid metabolism and ferroptosis-related genes, we constructed a prognostic signature capable of stratifying hepatocellular carcinoma patients into distinct high- and low-risk cohorts. These cohorts exhibit differential clinical outcomes, necessitating tailored therapeutic approaches. This model can provide more information for patient management and decision-making. Drug sensitivity analysis showed that high-risk patients may benefit from Dactolisib or Trametinib. It is worth noting that although high-risk patients have enhanced immune cell activity, their survival is worse due to frequent TP53 mutations and the characteristics of tumor stem cells. If immunotherapy is used, it is important to note that despite immune activation, high-risk tumors resist checkpoint blockade due to TP53 deletion, which suggests that we can adopt a combination strategy, such as the combination of ferroptosis inducers and anti-PD-1 inhibitors. The low-risk group is mainly characterized by an immunosuppressive microenvironment. PRDX1-targeted therapy can be tried, such as using Celastrol derivatives, which irreversibly inhibits its antioxidant function by alkylating Cys52.

Our study provides a foundational framework for understanding the lactylation-ferroptosis cross-talk. Moreover, while our study focused on the lactylation-ferroptosis axis, we acknowledge that this pathway operates within a broader metabolic network. Future work exploring its interplay with other metabolic and redox pathways will provide a more comprehensive understanding. While our *in vitro* experiments in MHCC-97H and SNU-449 cells provide crucial mechanistic evidence linking STMN1/PRDX1 to this axis, and we have strengthened the reliability of our conclusions by mutually validating key findings across these two distinct cell lines, we nevertheless acknowledge that these established cell line models cannot fully replicate the intricate tumor microenvironment, cellular heterogeneity, and immune interactions present *in vivo*. Consequently, the definitive causal role of STMN1/PRDX1 in regulating LDHA/GPX4 and driving tumor progression in a physiological context remains to be established. We have therefore incorporated plans to utilize patient-derived xenograft (PDX) models in our future research to robustly validate these findings in an *in vivo* setting that better preserves tumor-stroma interactions. Furthermore, the clinical applicability of our signature is currently constrained by its derivation from a single database (TCGA-LIHC), underscoring the need for external validation. Finally, the enticing therapeutic predictions generated by our model await rigorous preclinical testing. Future studies should aim to address these limitations by validating our model in larger, multi-center cohorts, incorporating more diverse experimental models, and elucidating the precise mechanistic links through techniques such as ChIP-seq to verify H3K18la enrichment at the STMN1/PRDX1 promoters.

In summary, we revealed that the combined effect of lactate metabolism and inhibition of ferroptosis is the key to HCC invasiveness. Four gene signatures can predict the survival of HCC patients, provide reference for targeted therapy, and provide a basis for subsequent research. These findings position STMN1 and PRDX1 not merely as potential predictive indicators for hepatocellular carcinoma (HCC) prognosis, but more critically, as central regulators governing its metabolic reprogramming and immune modulation. They can serve as a dual-channel synergy hub and be an ideal target for developing “metabolism-ferroptosis” co-targeted therapy and provide a new direction for promoting precision medicine for HCC.

## Data Availability

The original data presented in the study are openly available in The Cancer Genome Atlas (TCGA) database at https://portal.gdc.cancer.gov/, under the project name 'TCGA-LIHC'.
