# Supplementary material for: A lactylation-ferroptosis cross-talk gene signature predicts hepatocellular carcinoma prognosis and reveals STMN1/PRDX1 as therapeutic targets
Source: Front Immunol. 2025 Dec 1;16:1677089. doi: 10.3389/fimmu.2025.1677089 (PMC12702848; doi:10.3389/fimmu.2025.1677089)
Supplement: Supplementary file 1 [file Table1.doc]

The original uncropped blots are provided in Supplementary Materials.

Prior to antibody incubation, the membrane was sectioned into strips to allow simultaneous probing for different targets

All strips derive from the same membrane and experiment, with molecular weight markers aligned across strips.

Image contrast was appropriately adjusted for clarity.

Western blot analyses utilized two prestained protein ladders for molecular weight calibration:

1. Yaenzyme Prestained Ladder (Yaenzyme Biotechnology, China)
2. Zhuoyi Prestained Ladder (Zhuoyi Biotechnology, China)

All markers were from the same manufacturing lot per experiment.
