# Supplementary figures and images for: A lactylation-ferroptosis cross-talk gene signature predicts hepatocellular carcinoma prognosis and reveals STMN1/PRDX1 as therapeutic targets
Source: Front Immunol. 2025 Dec 1;16:1677089. doi: 10.3389/fimmu.2025.1677089 (PMC12702848; doi:10.3389/fimmu.2025.1677089)

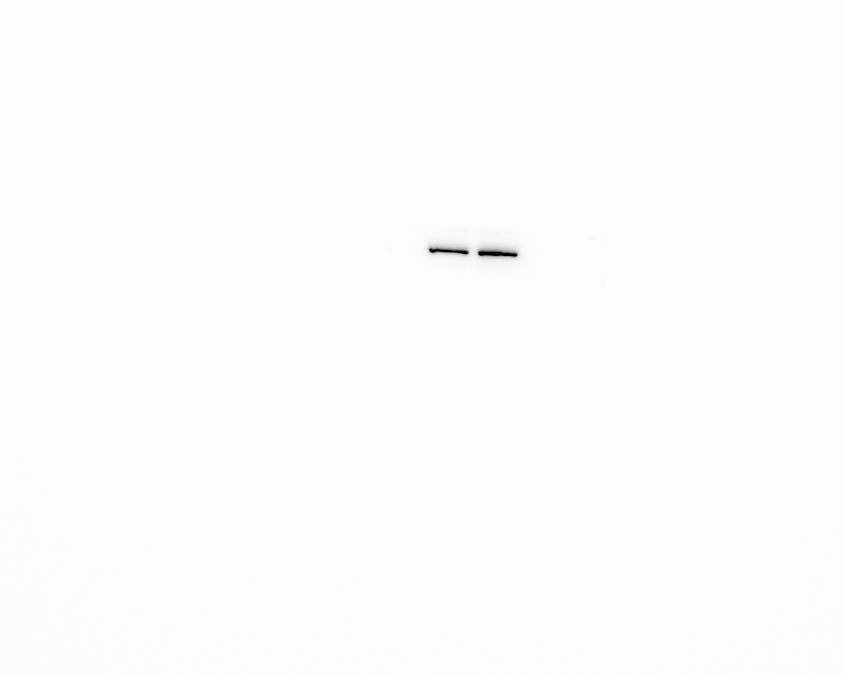

Supplement: Supplementary file 2 [file DataSheet1.zip › Supplementary_Western Blot_Knockdown of the PRDX1 in SNU-449/ACTIN.tif]

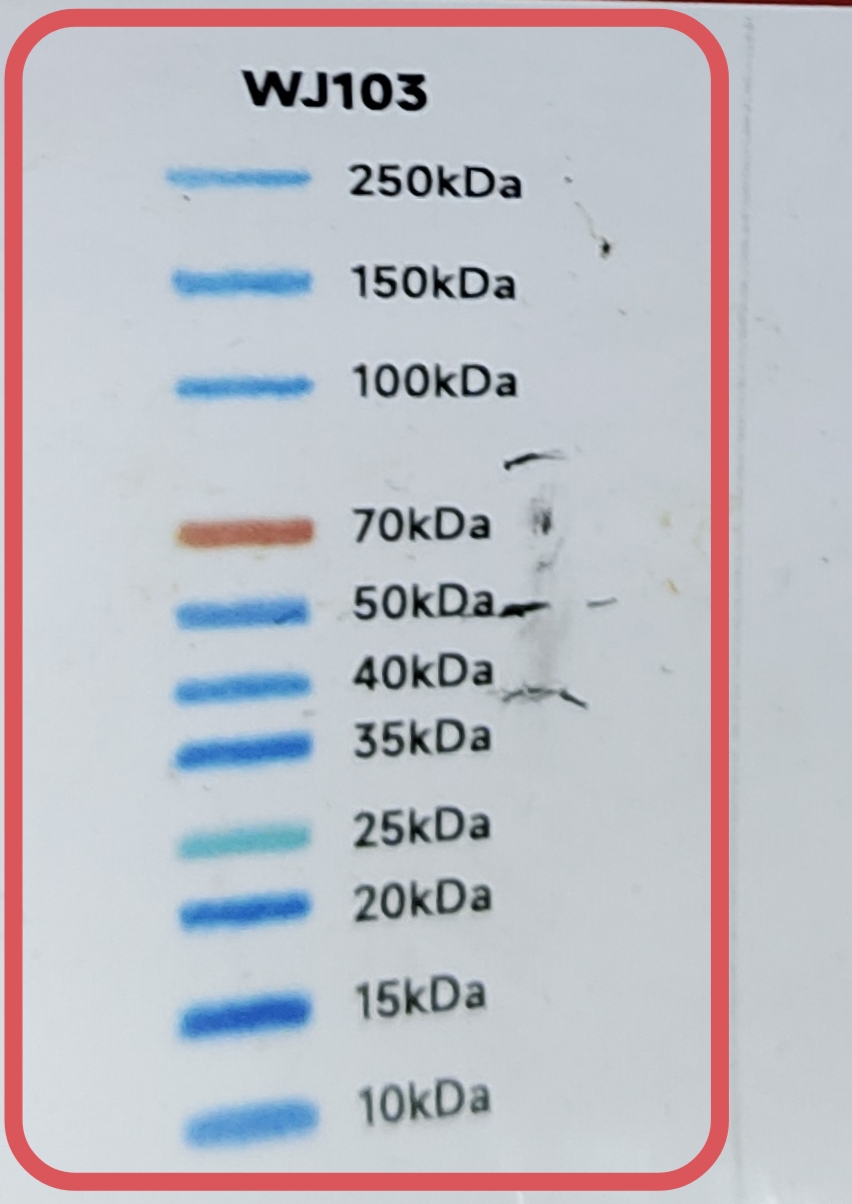

Supplement: Supplementary file 2 [file DataSheet1.zip › Supplementary_Western Blot_Knockdown of the PRDX1 in SNU-449/Molecular Weight Marker Reference (Yaenzyme, China).jpg]

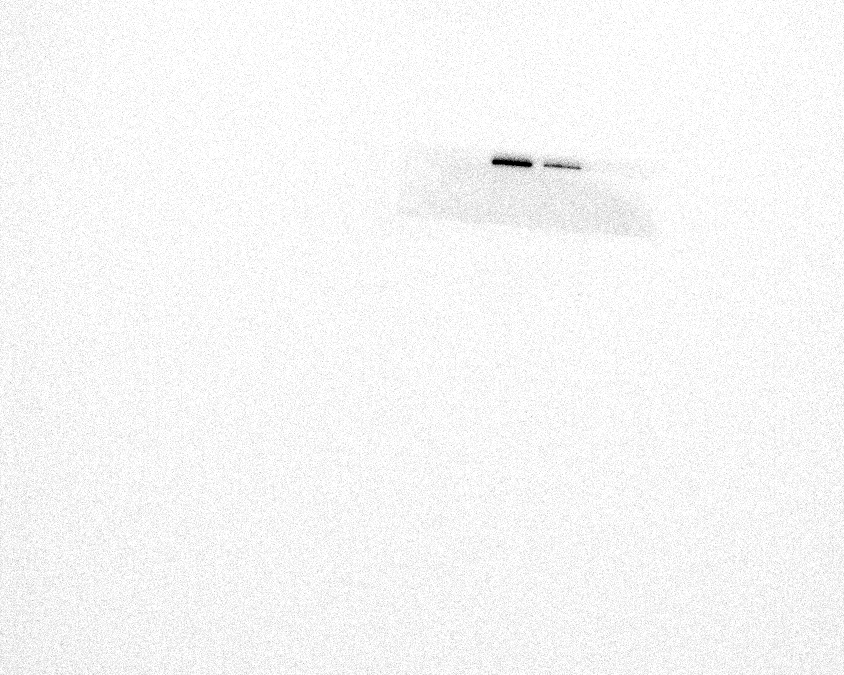

Supplement: Supplementary file 2 [file DataSheet1.zip › Supplementary_Western Blot_Knockdown of the PRDX1 in SNU-449/PRDX1.tif]

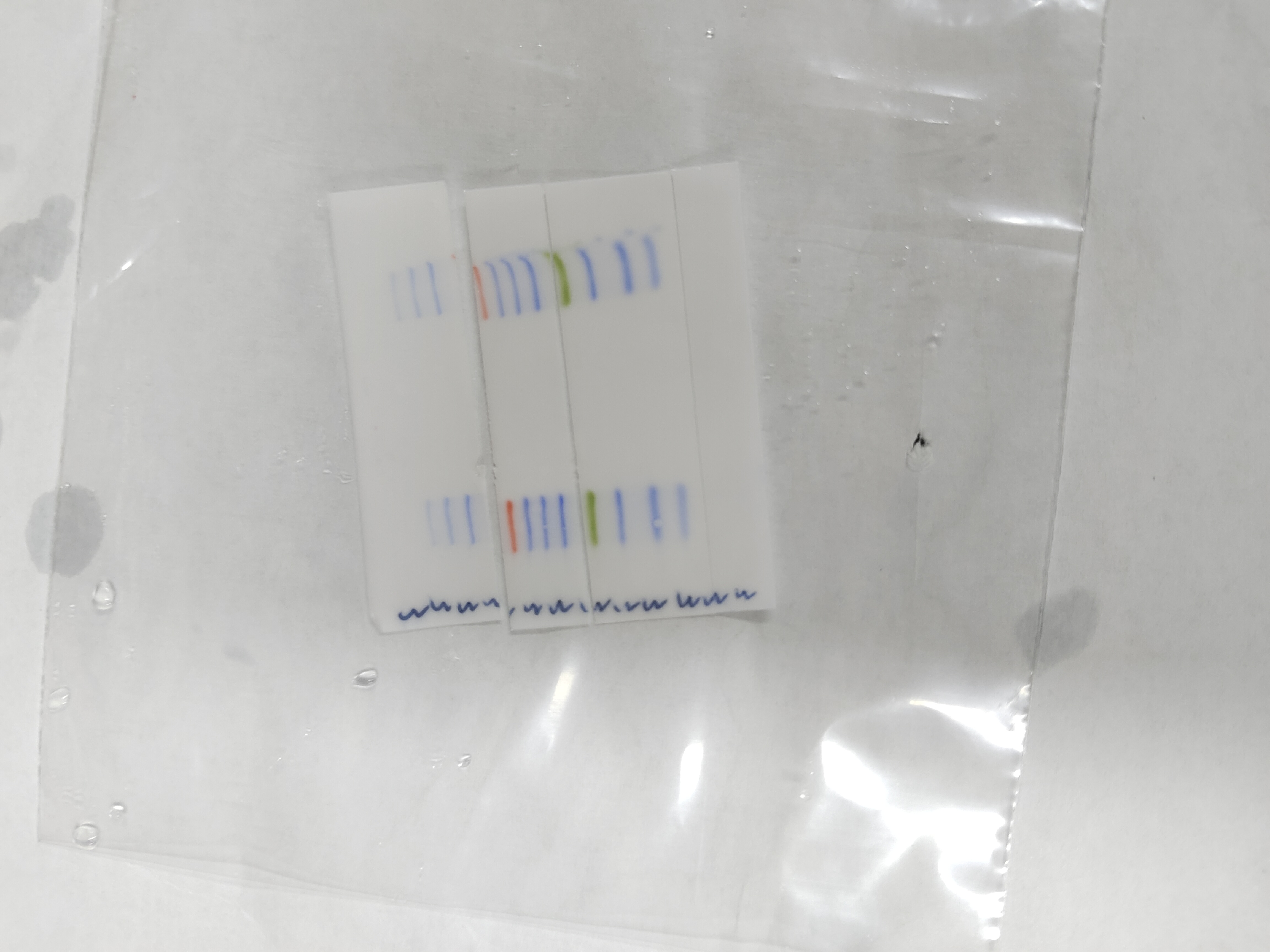

Supplement: Supplementary file 2 [file DataSheet1.zip › Supplementary_Western Blot_Knockdown of the PRDX1 in SNU-449/whole membrane after cut.jpg]

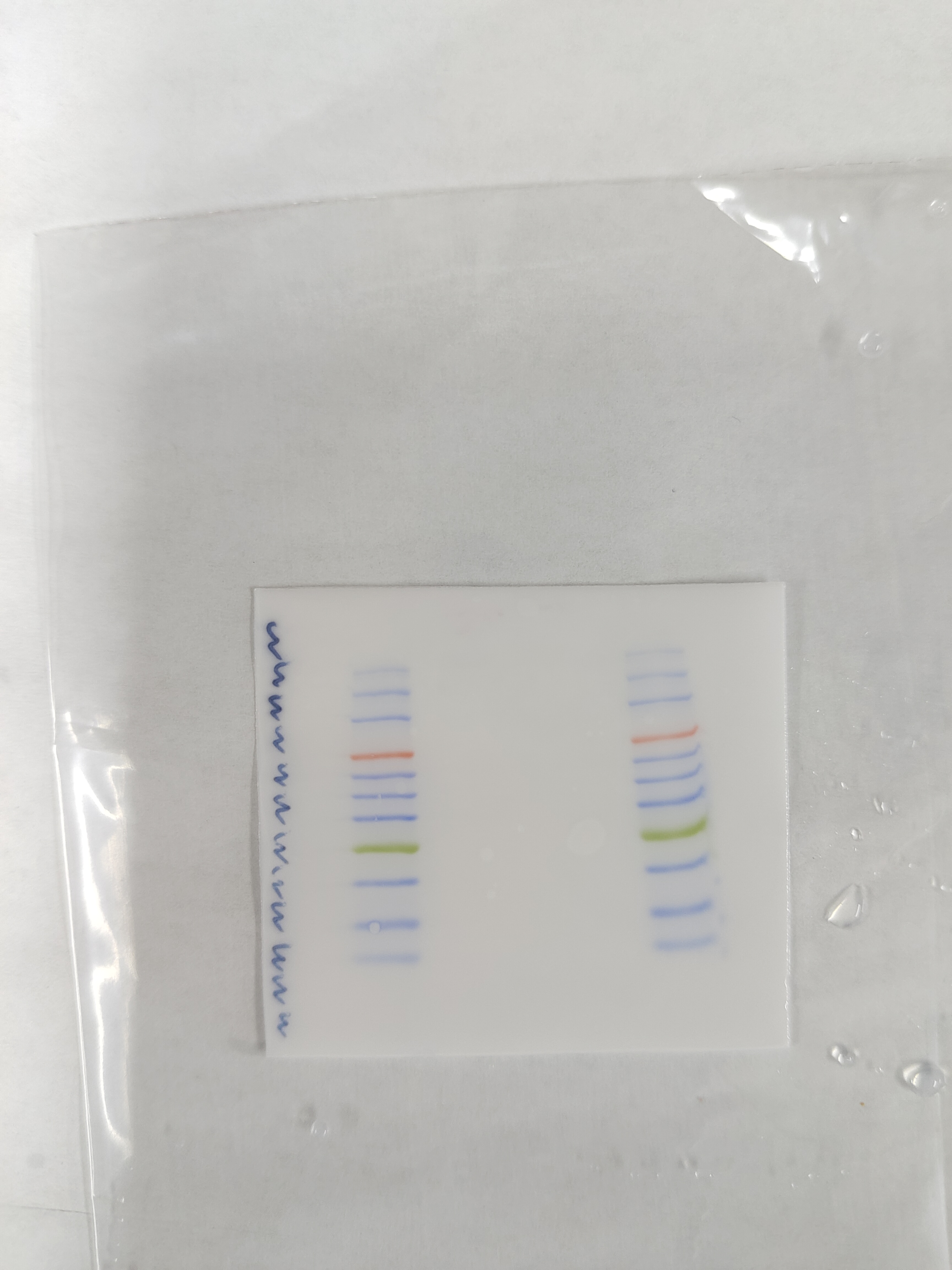

Supplement: Supplementary file 2 [file DataSheet1.zip › Supplementary_Western Blot_Knockdown of the PRDX1 in SNU-449/whole membrane.jpg]

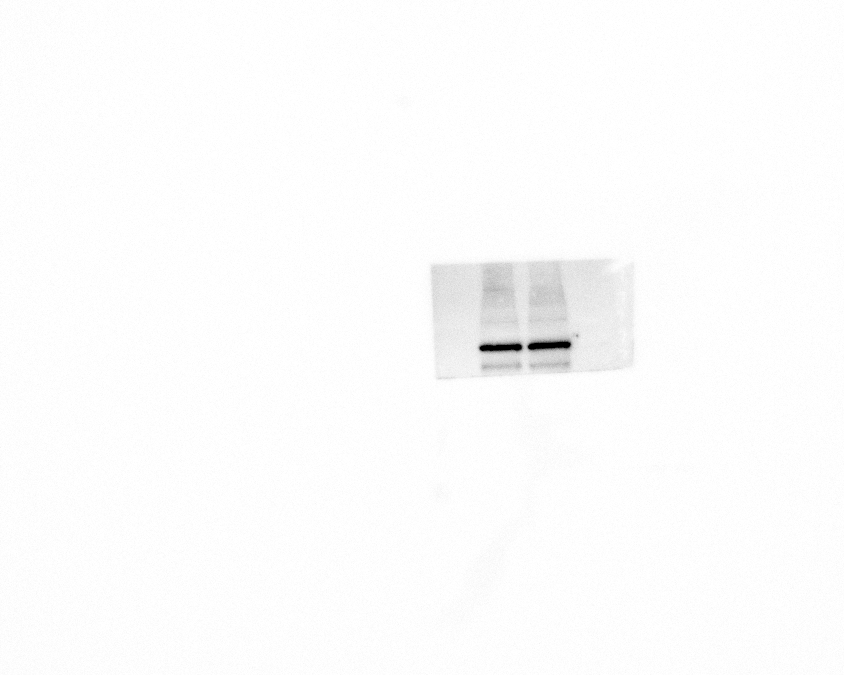

Supplement: Supplementary file 3 [file DataSheet2.zip › Supplementary_Western Blot_Knockdown of the PRDX1 in MHCC-97H/ACTIN.tif]

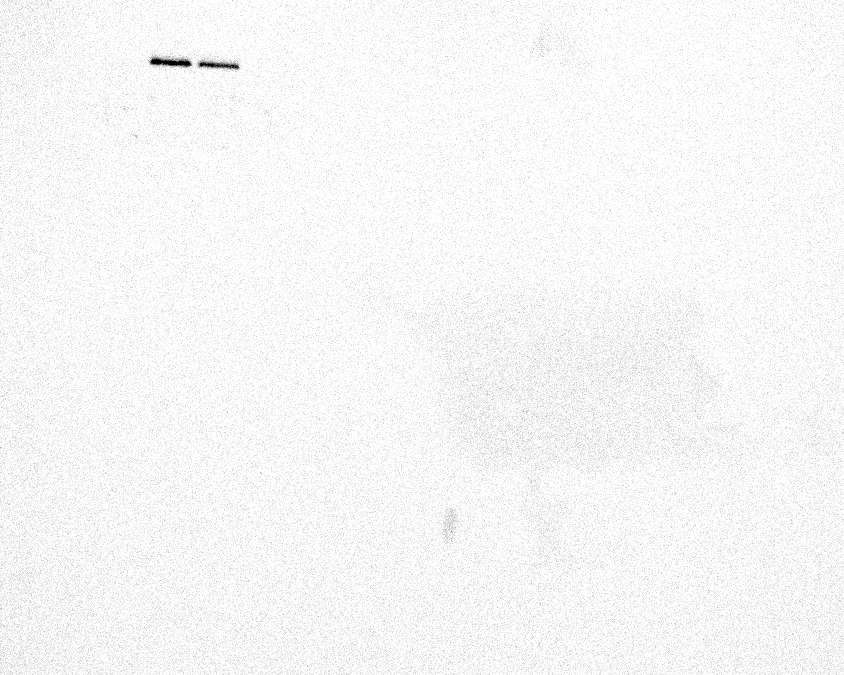

Supplement: Supplementary file 3 [file DataSheet2.zip › Supplementary_Western Blot_Knockdown of the PRDX1 in MHCC-97H/PRDX1.tif]

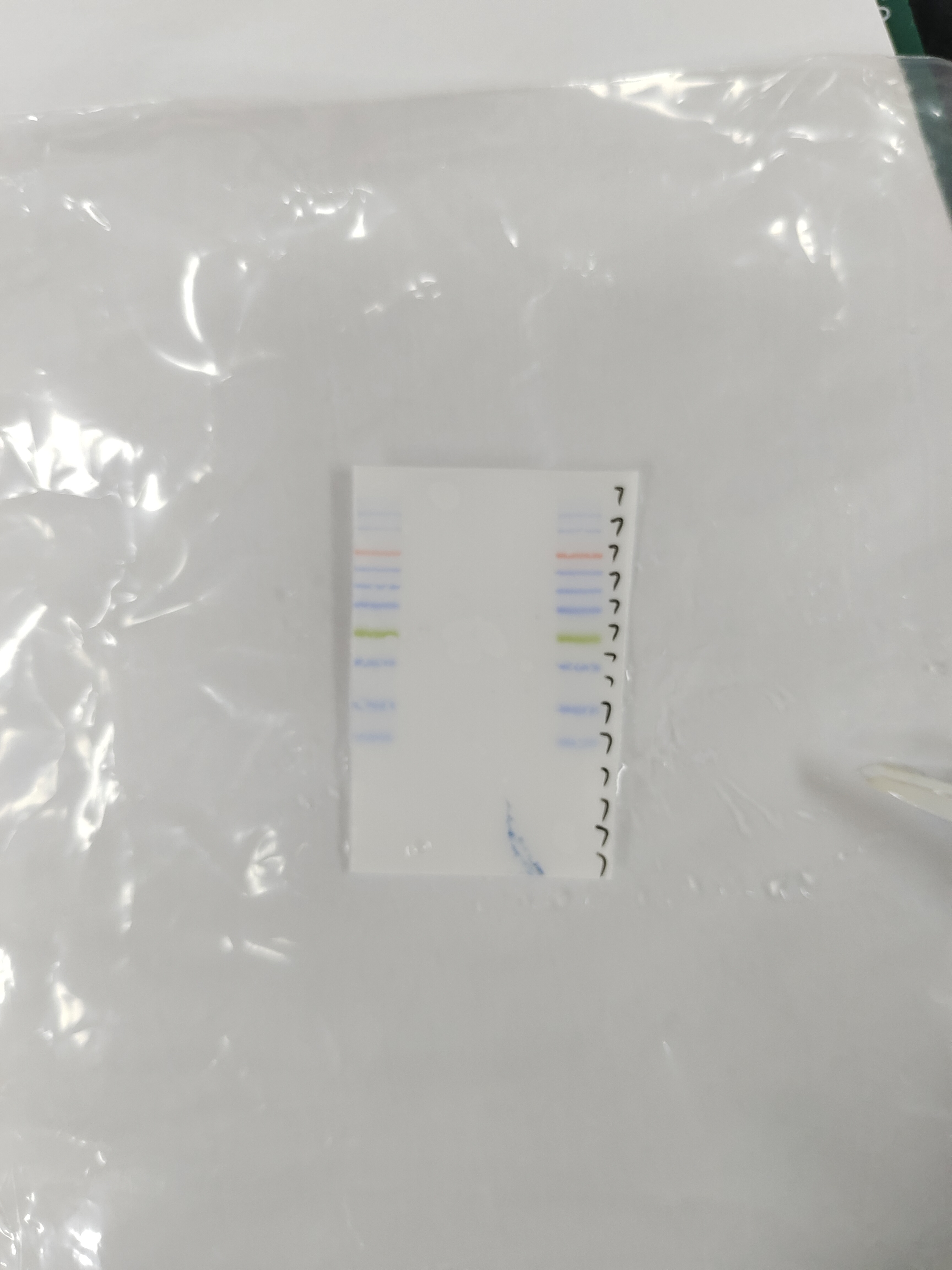

Supplement: Supplementary file 3 [file DataSheet2.zip › Supplementary_Western Blot_Knockdown of the PRDX1 in MHCC-97H/whole membrane after cut.jpg]

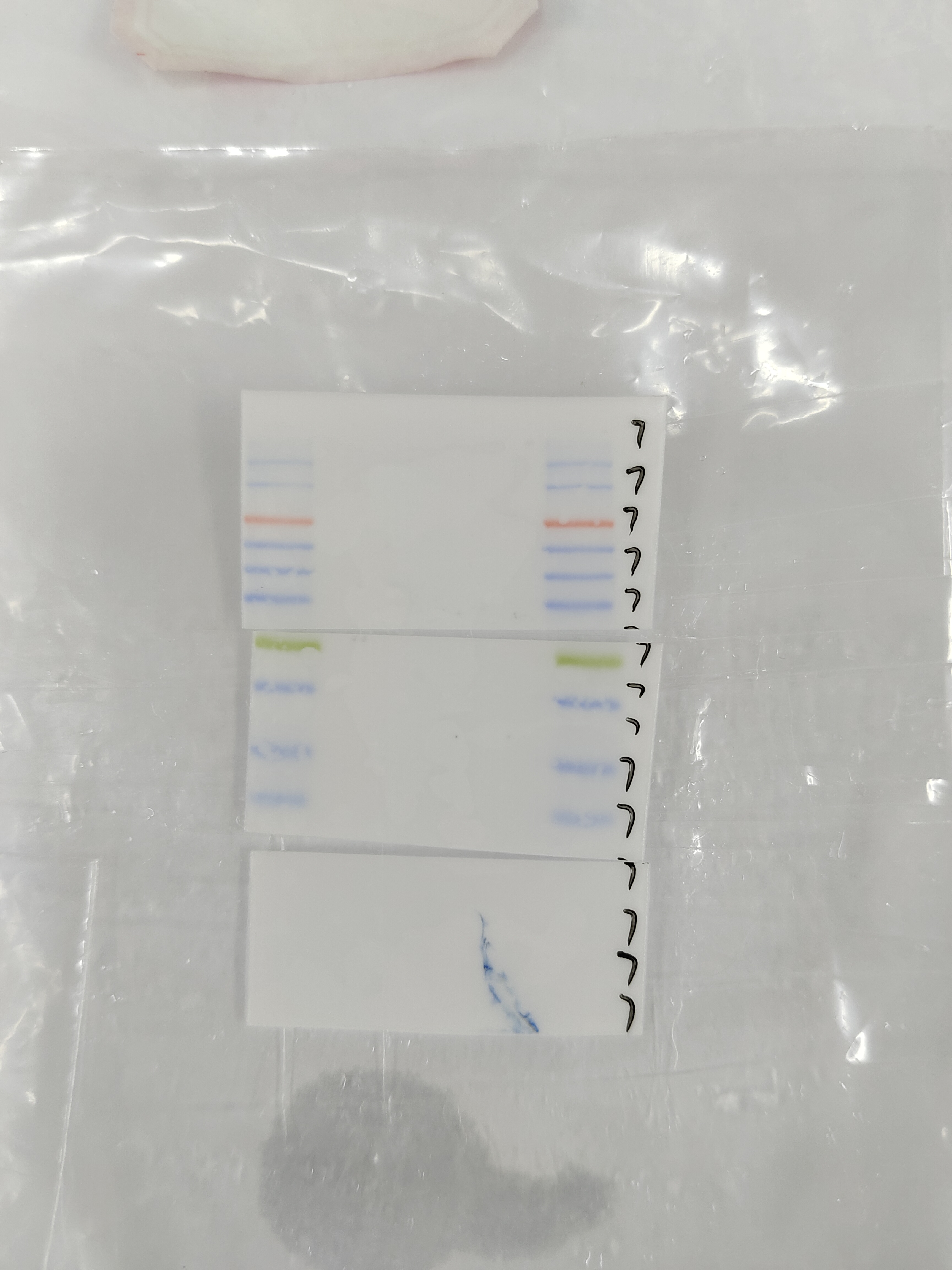

Supplement: Supplementary file 3 [file DataSheet2.zip › Supplementary_Western Blot_Knockdown of the PRDX1 in MHCC-97H/whole membrane.jpg]

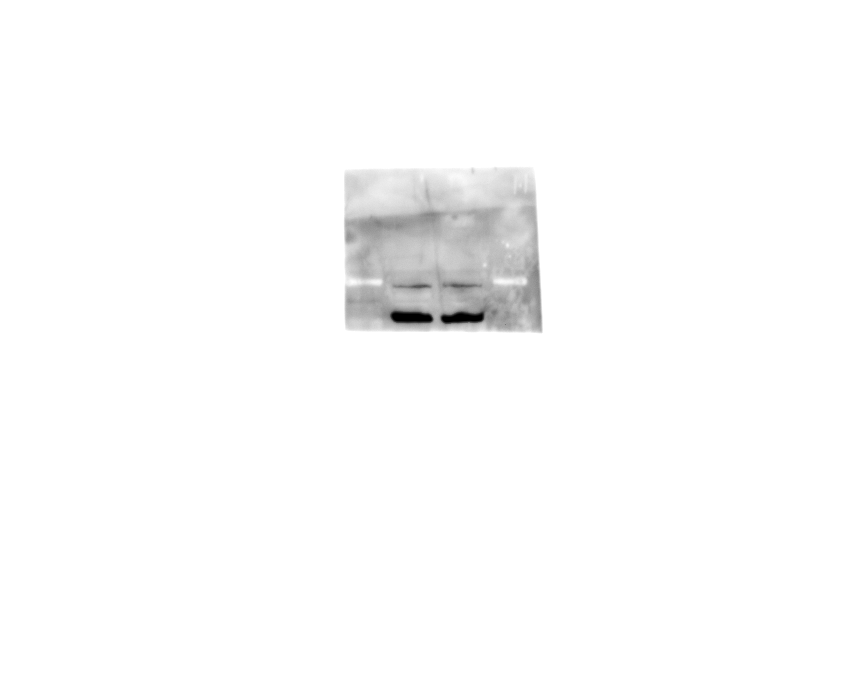

Supplement: Supplementary file 4 [file DataSheet3.zip › Supplementary_Western Blot_Knockdown of the STMN1 in MHCC-97H/ACTIN.tif]

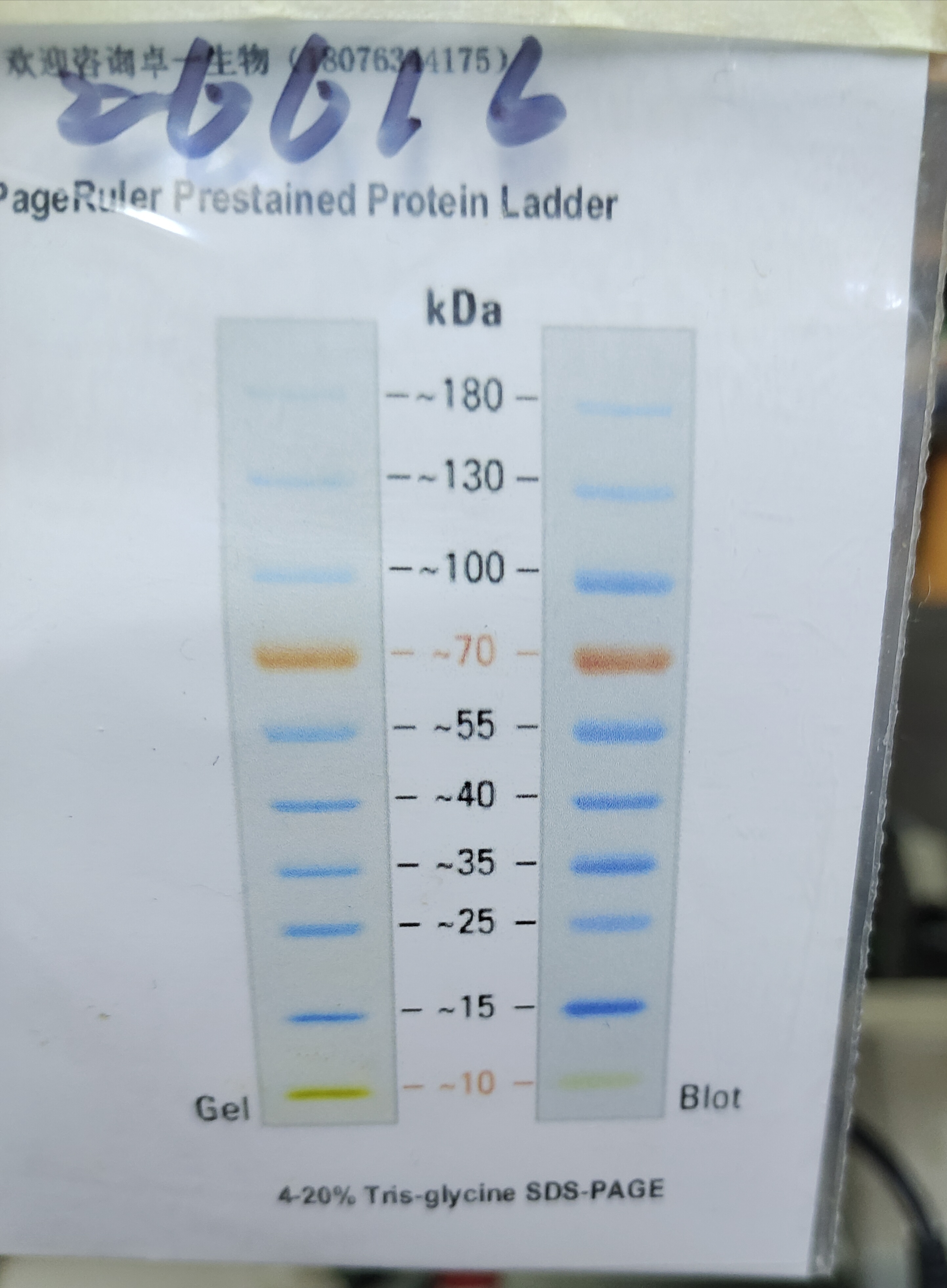

Supplement: Supplementary file 4 [file DataSheet3.zip › Supplementary_Western Blot_Knockdown of the STMN1 in MHCC-97H/Molecular Weight Marker Reference (Zhuoyi Biotechnology, China).jpg]

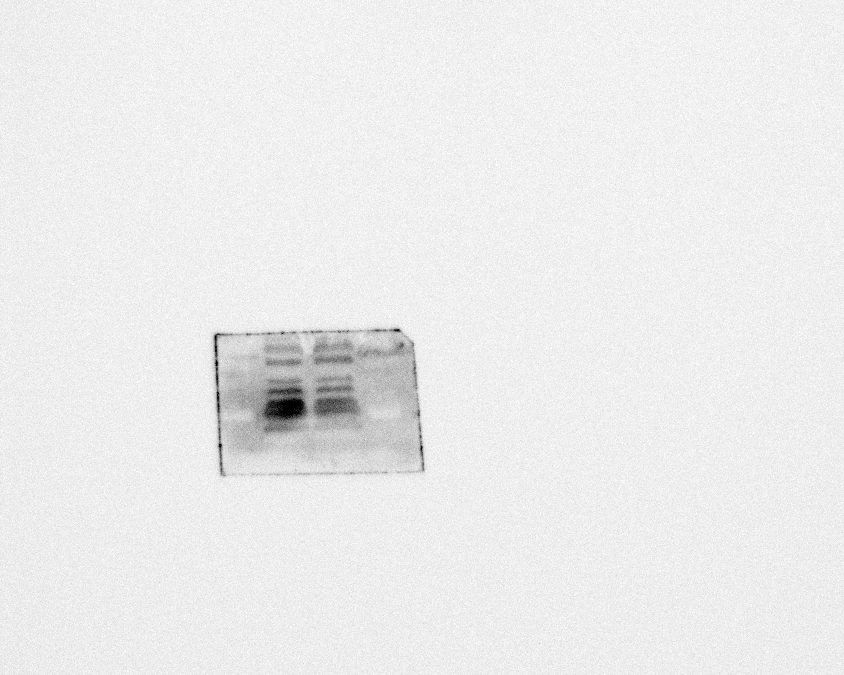

Supplement: Supplementary file 4 [file DataSheet3.zip › Supplementary_Western Blot_Knockdown of the STMN1 in MHCC-97H/STMN1.tif]

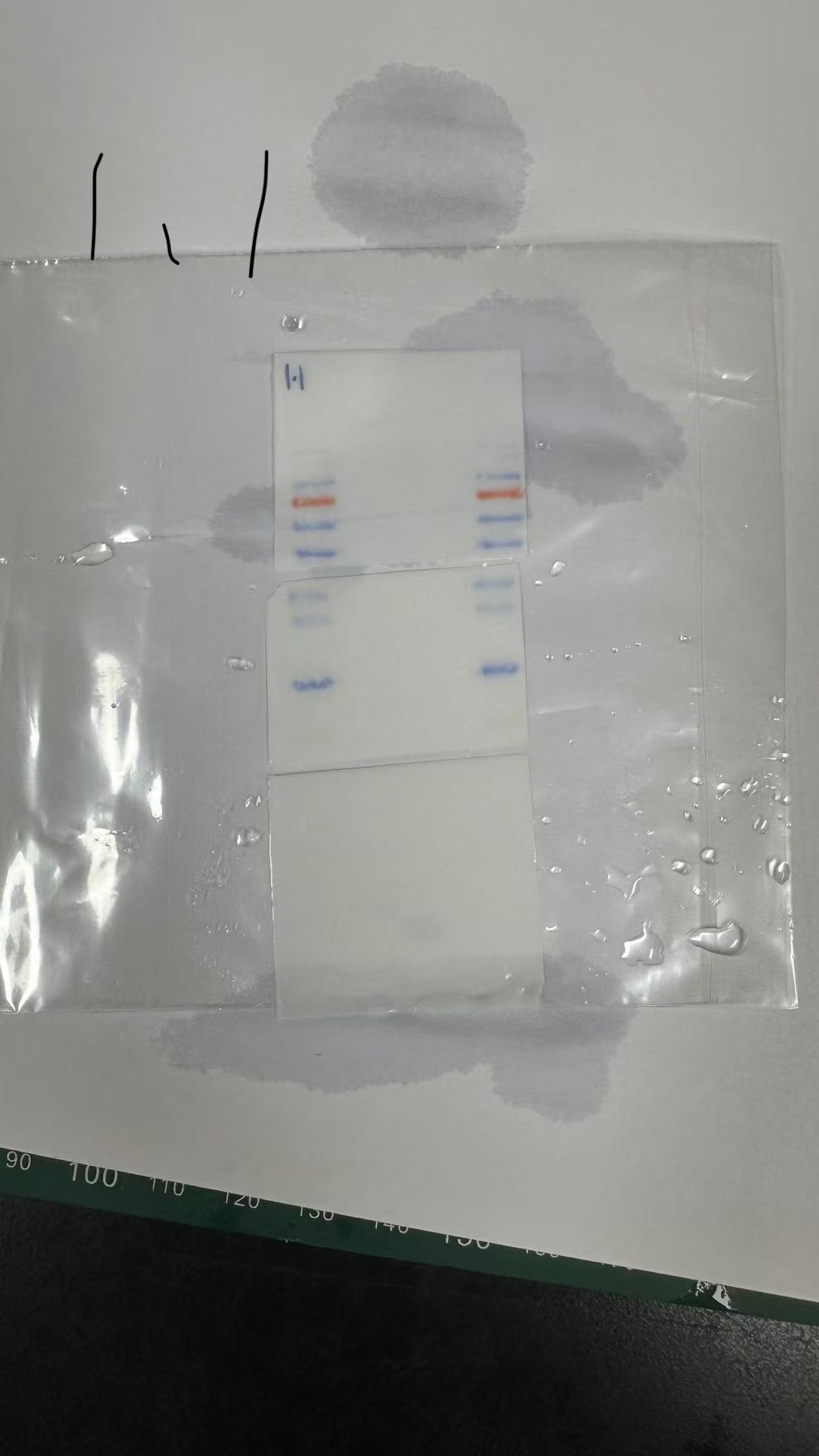

Supplement: Supplementary file 4 [file DataSheet3.zip › Supplementary_Western Blot_Knockdown of the STMN1 in MHCC-97H/whole membrane after cut.jpg]

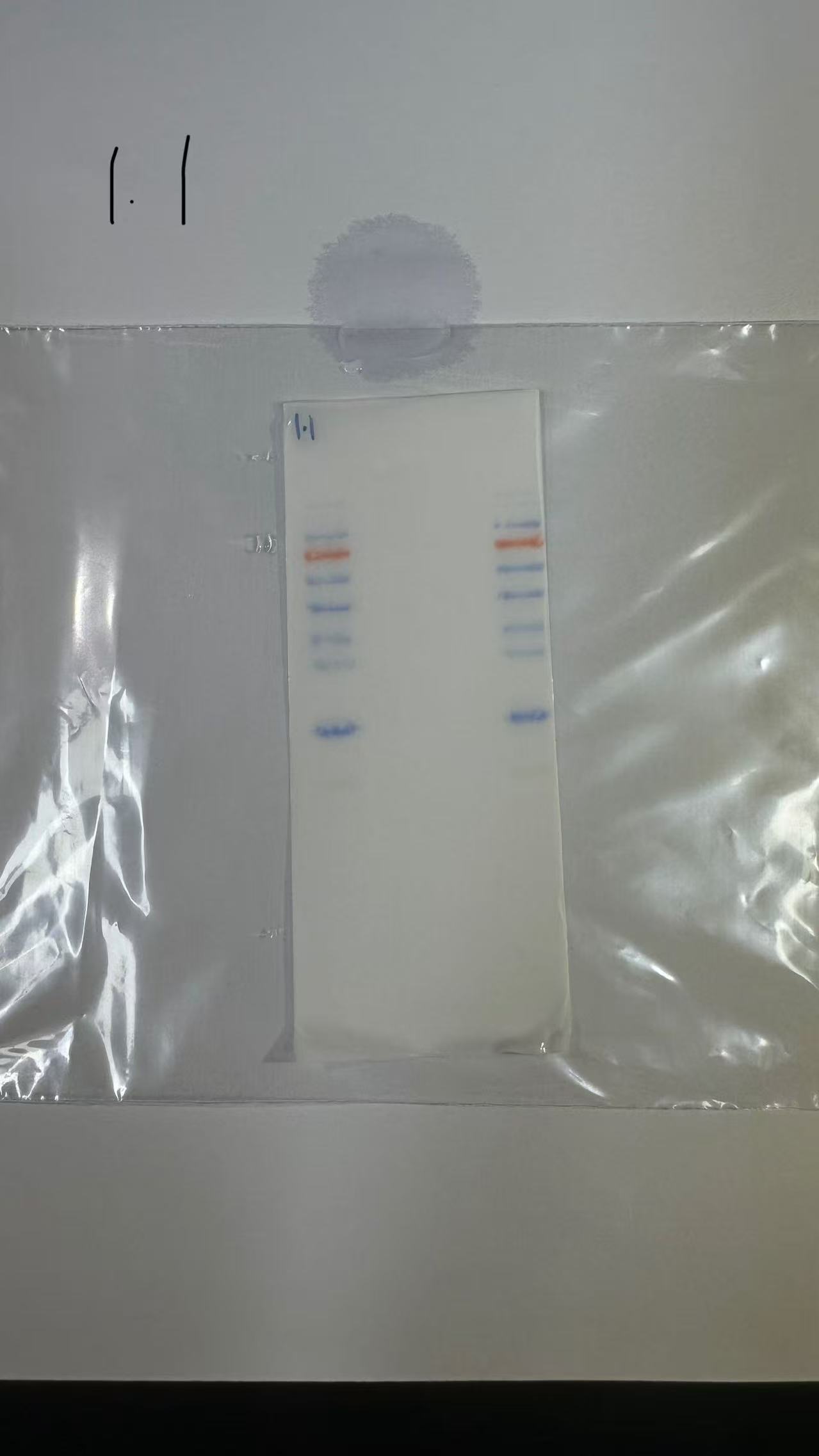

Supplement: Supplementary file 4 [file DataSheet3.zip › Supplementary_Western Blot_Knockdown of the STMN1 in MHCC-97H/whole membrane.jpg]

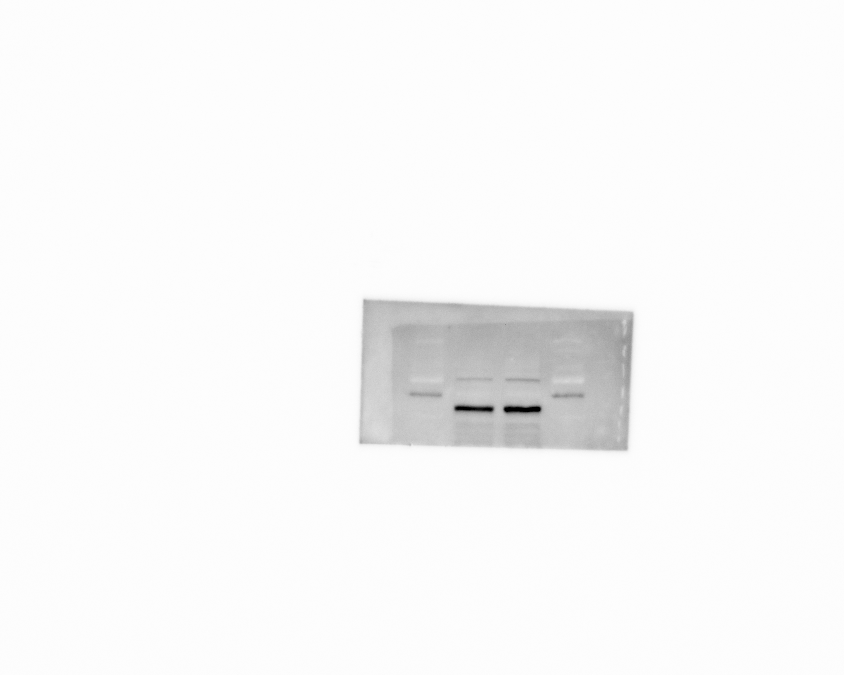

Supplement: Supplementary file 5 [file DataSheet4.zip › Supplementary_Western Blot_Knockdown of the STMN1 in SNU-449/ACTIN.tif]

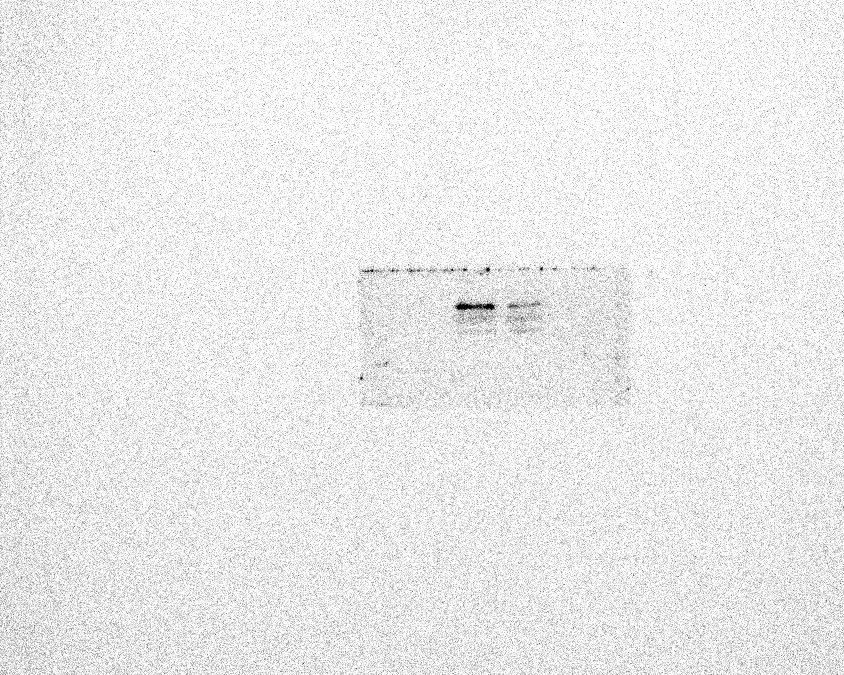

Supplement: Supplementary file 5 [file DataSheet4.zip › Supplementary_Western Blot_Knockdown of the STMN1 in SNU-449/STMN1.tif]

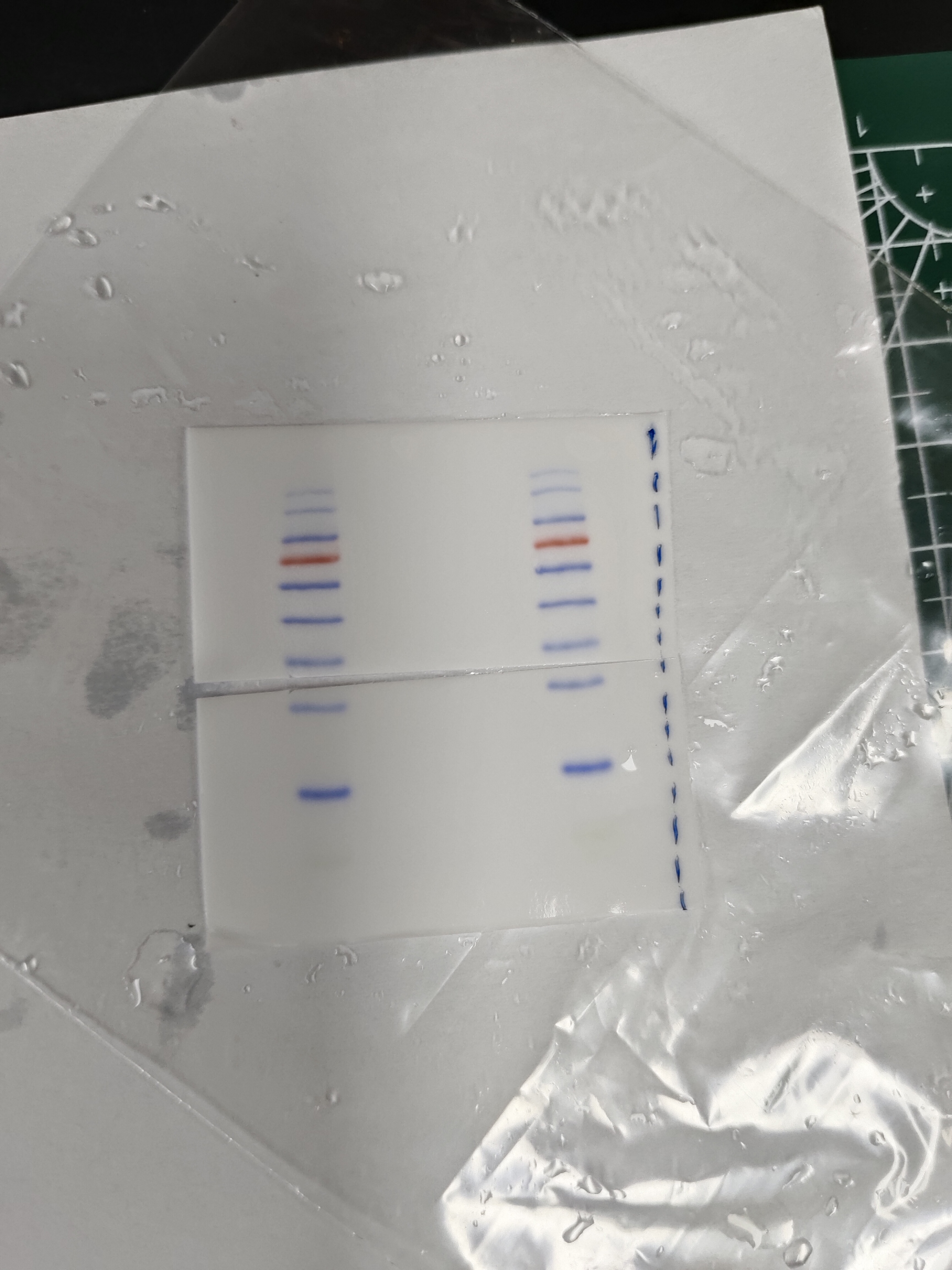

Supplement: Supplementary file 5 [file DataSheet4.zip › Supplementary_Western Blot_Knockdown of the STMN1 in SNU-449/whole membrane after cut.jpg]

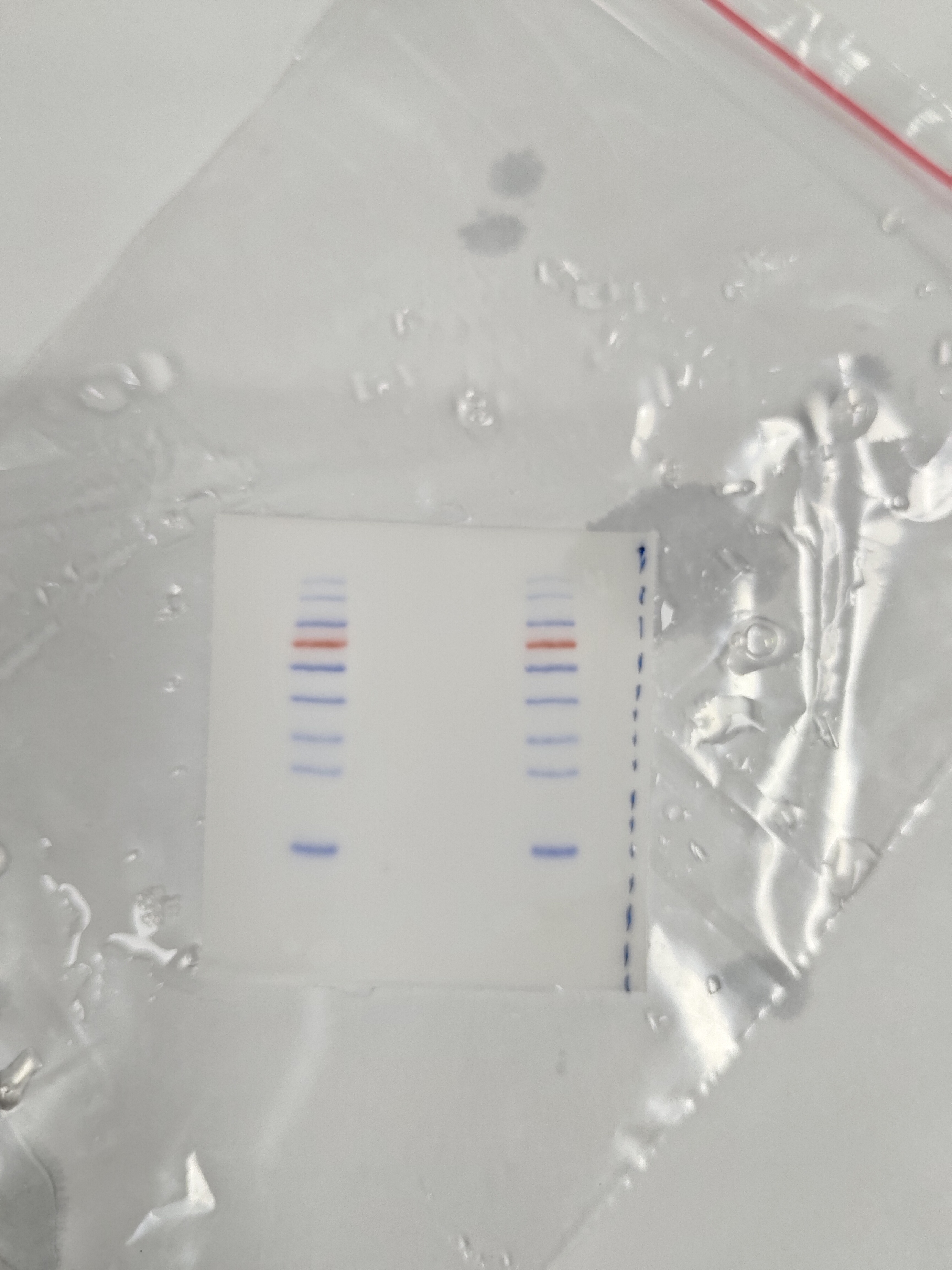

Supplement: Supplementary file 5 [file DataSheet4.zip › Supplementary_Western Blot_Knockdown of the STMN1 in SNU-449/whole membrane.jpg]

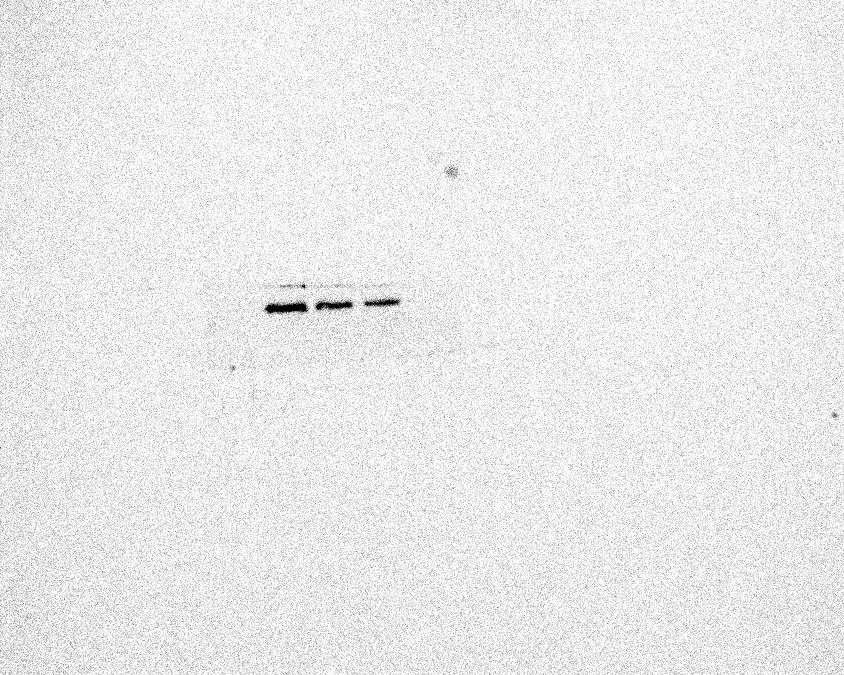

Supplement: Supplementary file 6 [file DataSheet5.zip › Supplementary_Western Blot_The decrease in the expression of proteins LDHA and GPX4 in MHCC-97H/GPX4.tif]

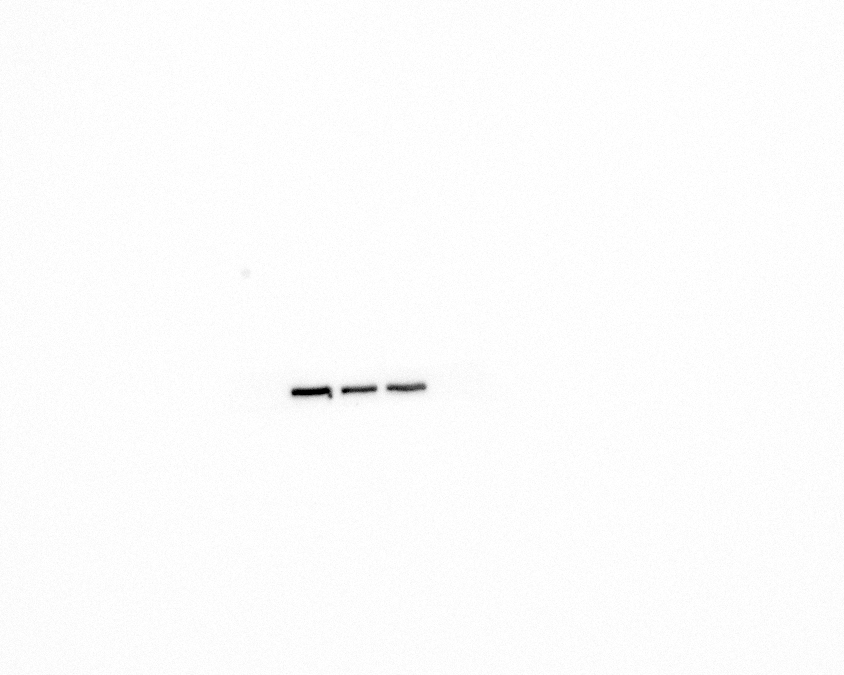

Supplement: Supplementary file 6 [file DataSheet5.zip › Supplementary_Western Blot_The decrease in the expression of proteins LDHA and GPX4 in MHCC-97H/LDHA.tif]

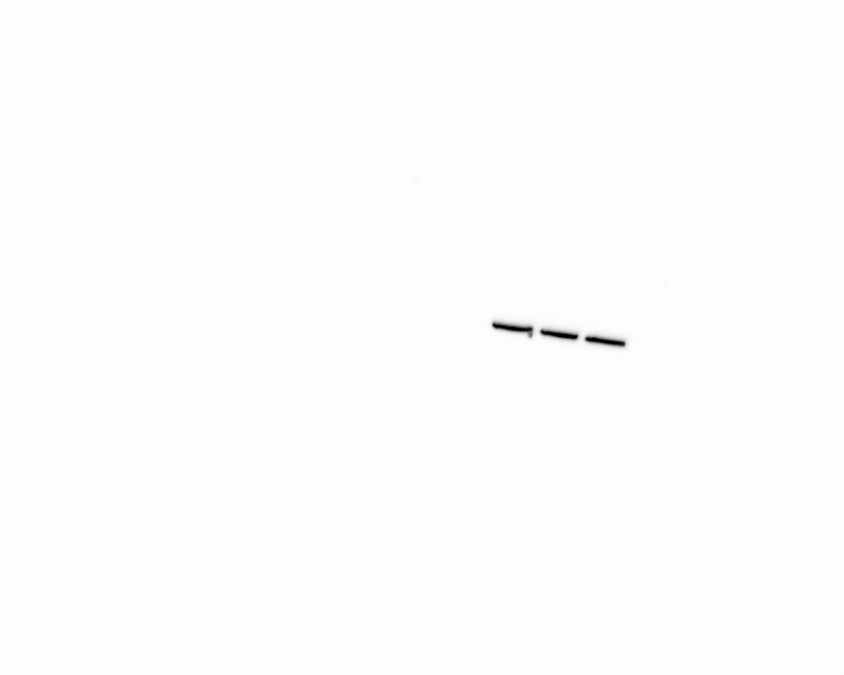

Supplement: Supplementary file 6 [file DataSheet5.zip › Supplementary_Western Blot_The decrease in the expression of proteins LDHA and GPX4 in MHCC-97H/TUBA.tif]

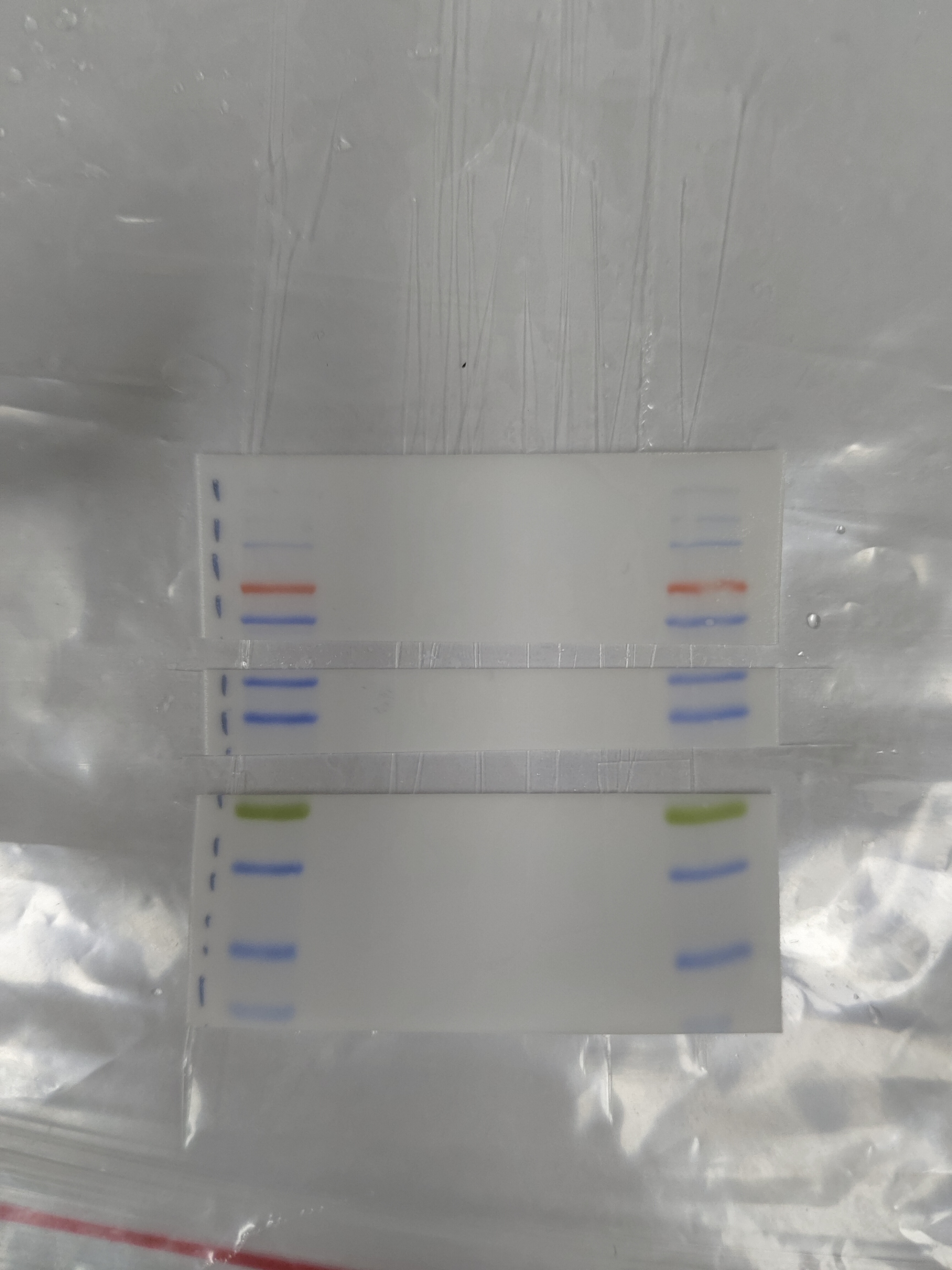

Supplement: Supplementary file 6 [file DataSheet5.zip › Supplementary_Western Blot_The decrease in the expression of proteins LDHA and GPX4 in MHCC-97H/whole membrane after cut.jpg]

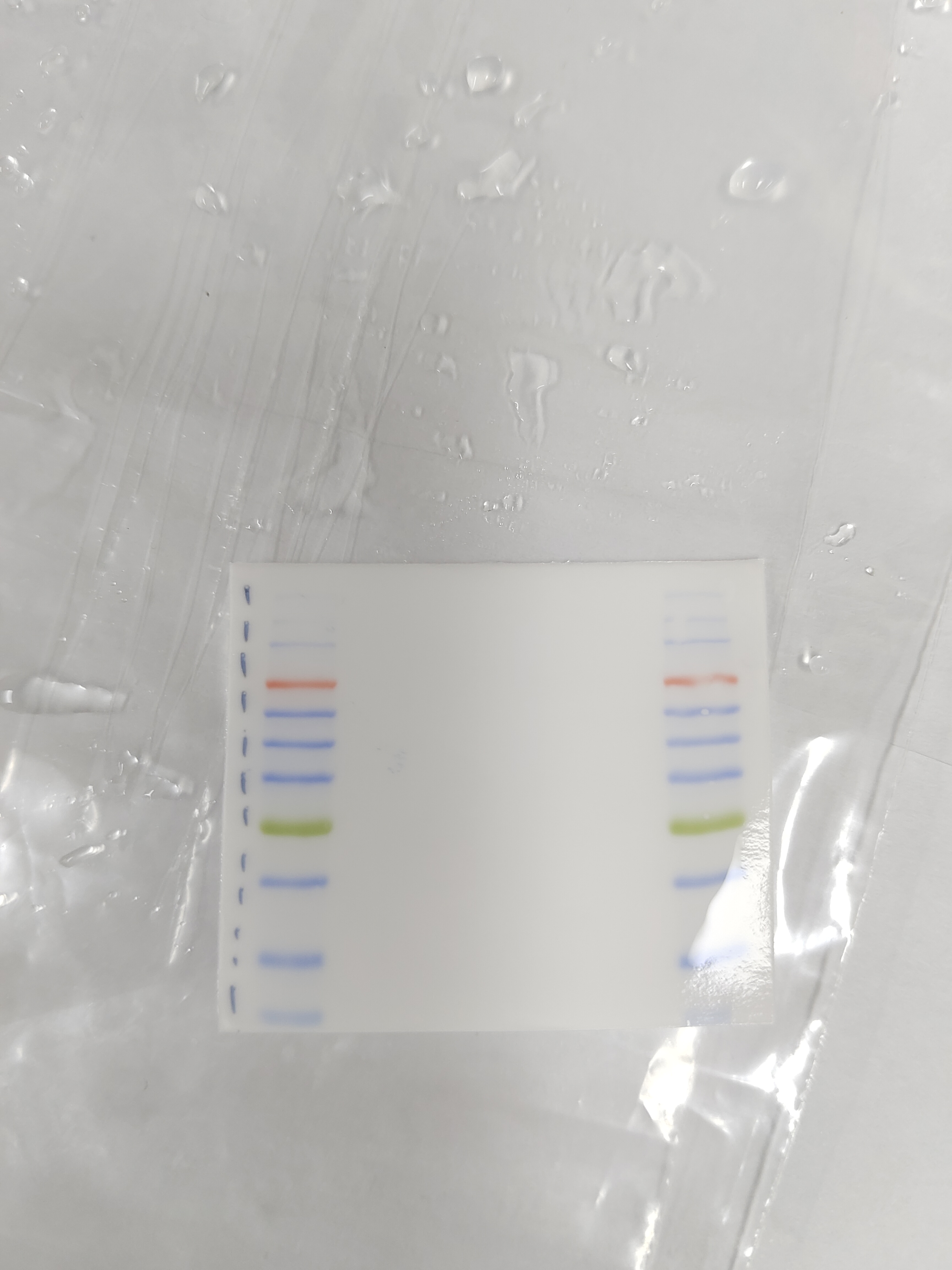

Supplement: Supplementary file 6 [file DataSheet5.zip › Supplementary_Western Blot_The decrease in the expression of proteins LDHA and GPX4 in MHCC-97H/whole membrane.jpg]

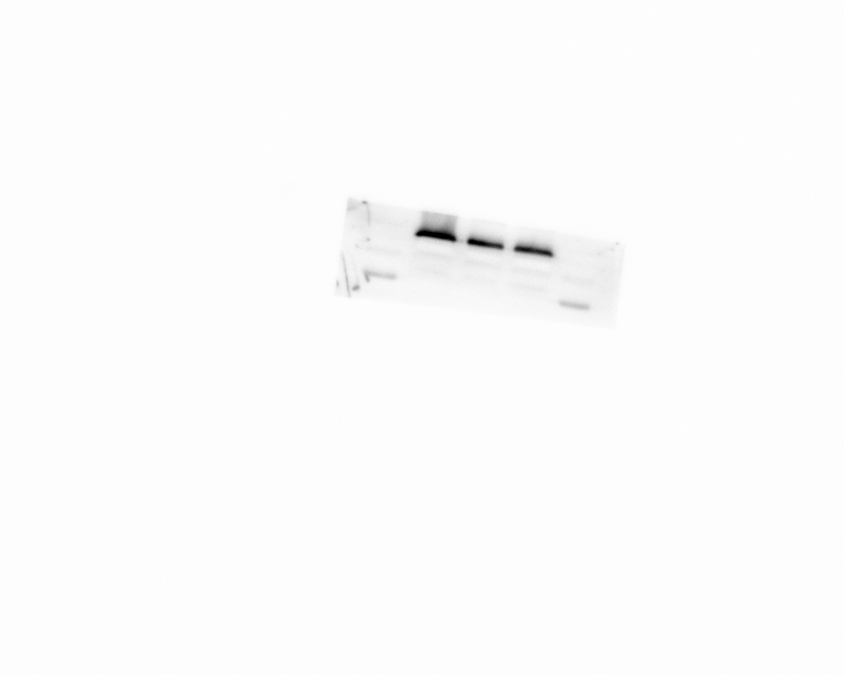

Supplement: Supplementary file 7 [file DataSheet6.zip › Supplementary_Western Blot_The decrease in the expression of proteins LDHA and GPX4 in SNU-449/GPX4.tif]

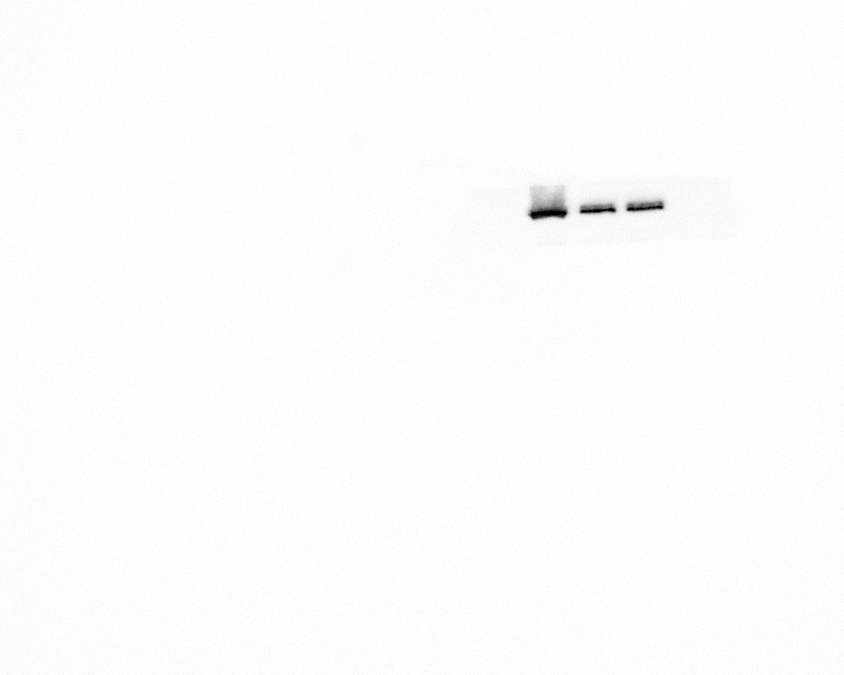

Supplement: Supplementary file 7 [file DataSheet6.zip › Supplementary_Western Blot_The decrease in the expression of proteins LDHA and GPX4 in SNU-449/LDHA.tif]

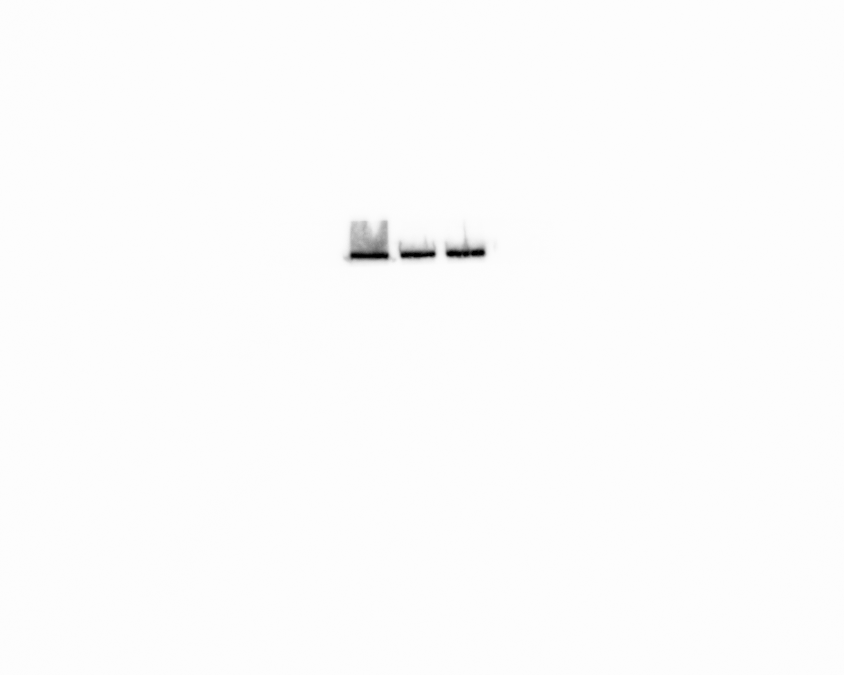

Supplement: Supplementary file 7 [file DataSheet6.zip › Supplementary_Western Blot_The decrease in the expression of proteins LDHA and GPX4 in SNU-449/TUBA.tif]

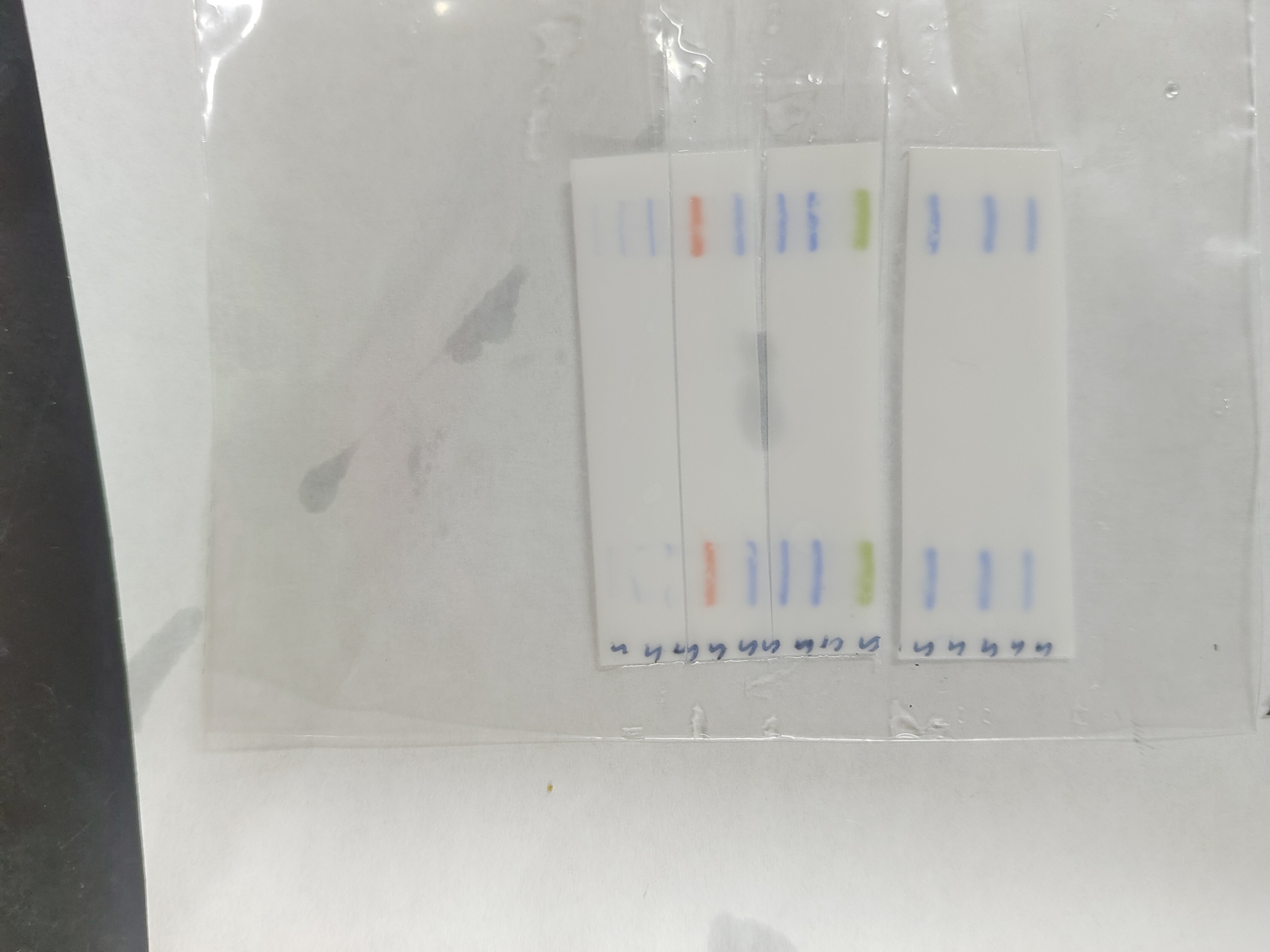

Supplement: Supplementary file 7 [file DataSheet6.zip › Supplementary_Western Blot_The decrease in the expression of proteins LDHA and GPX4 in SNU-449/whole membrane after cut.jpg]

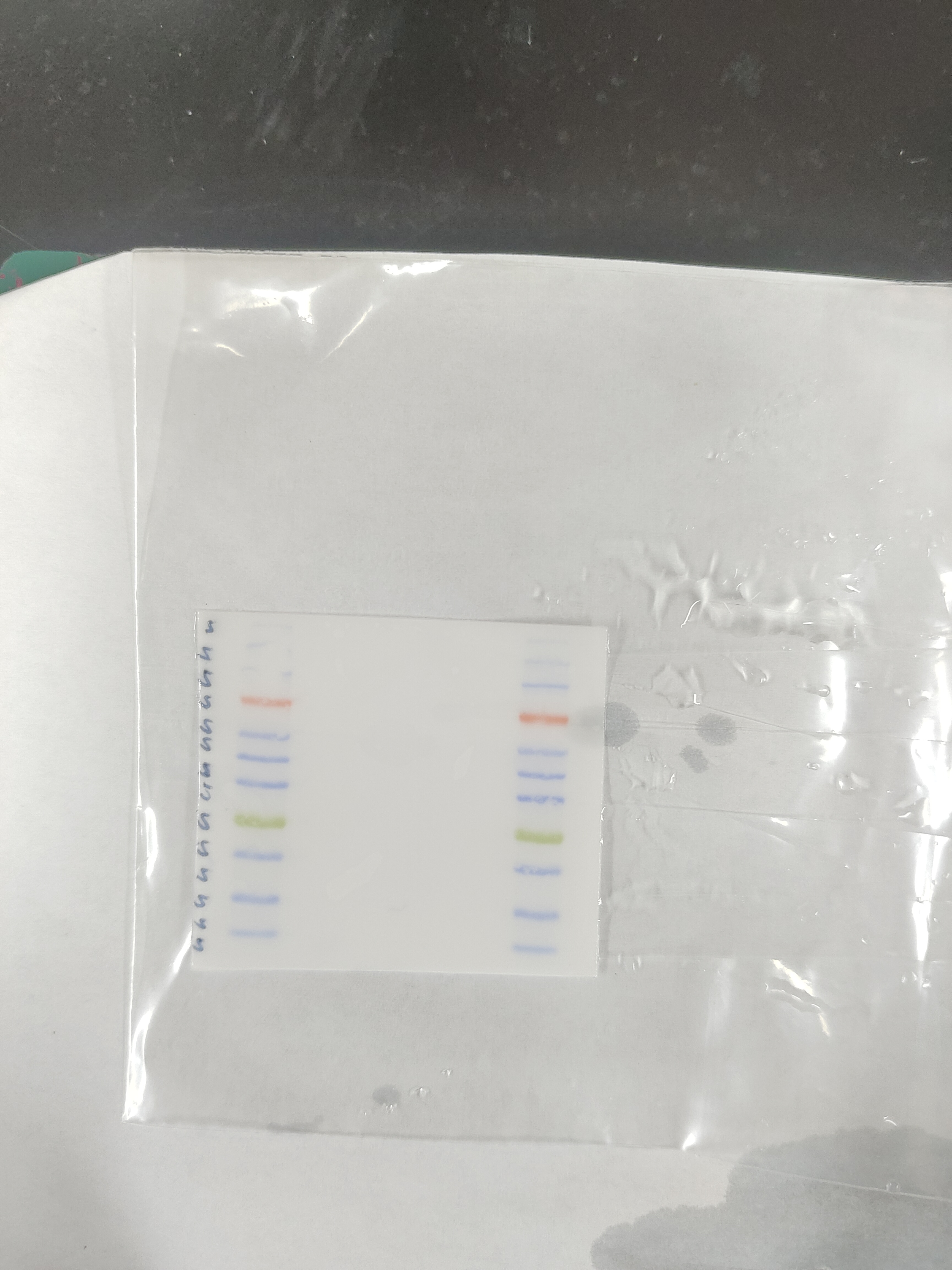

Supplement: Supplementary file 7 [file DataSheet6.zip › Supplementary_Western Blot_The decrease in the expression of proteins LDHA and GPX4 in SNU-449/whole membrane.jpg]
